# Comparing cost-effectiveness of short-course regimens for drug-resistant tuberculosis treatment in India

**DOI:** 10.1017/S0266462325100329

**Published:** 2025-07-21

**Authors:** Malaisamy Muniyandi, Balaji Ramraj, Sathishkumar Vadamalai, Sahil Abdul Salam, Bella Devaleenal, Jyoti Jaju, Chandrasekaran Padmapriyadarsini

**Affiliations:** 1 Department of Health Economics, ICMR-National Institute for Research in Tuberculosis, Chennai, India; 2 Department of Clinical Research, ICMR-National Institute for Research in Tuberculosis, Chennai, India; 3 iDFFEAT TB Project, International Union Against Tuberculosis and Lung Disease, New Delhi, India

**Keywords:** TB, DR-TB, cost-effectiveness, BPaL, BPaLM, BPaLC, BEAT, mBPaL, shorter regimen

## Abstract

**Background:**

Short-course regimens are currently explored to improve multidrug-resistant tuberculosis effects, reduce costs, as well as enhance patient adherence. Currently, we are determining the most cost-effective shorter regimen out of seven short-course regimens (6–9 months) to treat drug-resistant tuberculosis (DR-TB) compared to the current standard of care (SoC) 9- to 11-month regimen.

**Methods:**

Cost-effectiveness of various short-course DR-TB treatment regimens, namely BEAT, BPaL, BPaLM, BPaLC, mBPaL1, mBPaL2, and mBPaL3, was compared to the current SoC in India. Decision tree model was used from a health system perspective. The information on various costs – such as preinvestigations, regimens, adverse drug reactions (ADRs) management, inpatient treatment – and on effect – such as clinical outcomes and ADRs – was collected from different published sources. It estimated costs, quality-adjusted life years, and incremental cost-effectiveness ratios (ICERs). Sensitivity analyses were performed to validate outcomes against the willingness-to-pay threshold.

**Results:**

When all the short-course regimens were compared with the current SoC regimen, the ICERs were ₹5,385, ₹2,014, ₹2,008, ₹2,435, ₹1,462, ₹1,159, and ₹1,895 for BEAT, BPaL, BPaLM, BPaLC, mBPaL1, mBPaL2, and mBPaL3, respectively. Among the short-course regimens, mBPaL2 is the dominant strategy, and mBPaL1 has extended dominance. For all Bedaquiline-containing regimens, the cost of the drug is a crucial factor in determining cost effectiveness. The cost-effectiveness acceptability curve showed that all shorter regimens were 100 percent cost-effective.

**Conclusion:**

The implementation of Bedaquiline-based regimen to treat DR-TB has become more effective, shorter in duration, and less burdensome to the health system.

## Introduction

Tuberculosis (TB) remains a significant global health challenge, with an estimated 10.8 million people developing TB and 1.3 million deaths reported in 2023, making it the leading cause of mortality among communicable diseases worldwide (1). India accounts for 27 percent of the world’s TB cases in 2023 (1). Despite advances in medical science, TB continues to claim millions of lives annually. Pulmonary TB, one of the most common forms of TB, poses a persistent threat, particularly when it evolves from drug-sensitive TB (DS-TB) to drug-resistant TB (DR-TB), including multidrug-resistant, rifampicin-resistant TB (MDR/RR-TB), pre-extensively DR-TB, and extensively drug-resistant TB (XDR-TB). According to the World Health Organization (WHO), MDR-TB is a form of TB disease caused by a strain of *Mycobacterium tuberculosis* complex that is resistant to rifampicin and isoniazid. Worldwide, an estimated 400,000 people developed MDR/RR-TB, but only 175,923 people were diagnosed and started on treatment in 2023, which is significantly lower than in previous years (1).

India has the highest burden of MDR-TB, bearing 27 percent of the global burden, with an estimated 130,000 people developing the disease. A total of 79,000 MDR/RR-TB cases were notified in the country; however, only 36 percent were diagnosed, and 34 percent started on treatment, which is significantly lower than expected (2). The challenges for this gap include limited access to diagnostic tools, inadequate human resources, lack of funding, and a healthcare system that is not well equipped with adequate laboratory capacity for diagnosing MDR TB patients (3). Consequently, MDR-TB became a major problem, arising from the poor management of DS-TB and DR-TB. India’s TB control program is operated by the Ministry of Health and Family Welfare, Government of India, as National Tuberculosis Elimination Programme (NTEP). It follows the WHO’s recommended strategies and it has scaled up free diagnostic and treatment services nationwide, integrating both public and private sectors. The program is primarily funded by domestic government budget. However, India’s per capita spending on TB remains significantly lower compared to other middle-income countries, such as South Africa and Brazil, which invest more in patient-centered care and TB-specific social support systems. This disparity in funding and resource allocation contributes to persistent challenges in case detection, treatment adherence, and MDR-TB management in India.

MDR-TB is treatable, but it is very expensive and requires a long duration of treatment. This treatment is, however, both costly and time-consuming. The standard treatment plan involves a rigorous 8-month initial phase followed by a 12-month continuation phase, totaling 20 months (4–6). Globally, the treatment success rate for MDR-TB is around 63 percent (7). Unfortunately, in India, the success rate is only 46 percent, whereas in developed countries, it is 70.6 percent in the United Kingdom and 77 percent in Russia (8). A meta-analysis of 12,030 patients from 25 countries in 50 studies reported 7,346 (61 percent) had treatment success, 1,017 (8 percent) had failure or relapse, and 1,729 (14 percent) died (9). The reasons for low success rate are due to the lengthy and harsh nature of the treatment, which can lead to poor patient adherence (10). The other reason could be the expensive nature of treatment. It was reported that the treatment cost for MDR-TB was $5723, and it involves daily injectables in the intensive phase, management of side effects, investigations, and prolonged days of work lost (11). To combat this global health threat, we need innovative strategies to make MDR-TB treatment more effective. These strategies should focus on reducing treatment duration, lowering costs, and improving patient experience (12;13).

Globally, several trials, such as TB-TRUST, endTB, endTB-Q, Nix-TB, ZeNix Trials, TB-PRACTECAL, NExT, and STREAM Trials, are being conducted to estimate the clinical effectiveness of short-course DR-TB treatment regimens (14–21). These regimens showed promising results, offering a shorter, injectable-free treatment option for DR-TB patients (15;16;21). Researchers in India are actively engaged in developing and accessing shorter treatment regimens for DR-TB. These regimens aim to reduce the strain on the health system and patients by reducing treatment time and optimizing resources. Shorter regimens are also expected to enhance patient compliance, reduce the likelihood of adverse effects, and lower the overall costs associated with prolonged treatment (13;22). However, the success of these shorter regimens depends on their adaptability to various healthcare settings. It also relies on the commitment of healthcare providers to adhere to updated treatment protocols.

This study focuses on evaluating the cost-effectiveness of various short-course treatment regimens, which are currently being tested in India to treat DR-TB, including BEAT, BPaL, BPaLM, BPaLC, and Modified BPaL (mBPaL1, mBPaL2, and mBPaL3) regimens, as compared to the current standard of care (SoC) 9- to 11-month regimen. It estimates the incremental cost incurred from shorter regimens to treat DR-TB in comparison with the SoC and also estimates incremental cost-effectiveness ratio (ICER) and quality-adjusted life years (QALYs) gained by the shorter regimens. The findings aim to provide critical insights for policymakers to adopt the most cost-effective therapy for programmatic use, ensuring effective and sustainable TB control within the public health system. By considering the cost-effectiveness from the health system perspective, this research will highlight the long-term economic impact on the NTEP in India and inform strategies to reduce the financial burden on TB patients facing catastrophic expenses. Furthermore, successful implementation of cost-effective regimens can strengthen TB control efforts, contributing to global TB elimination goals and enhancing overall public health outcomes. Addressing the economic implications of TB treatment is crucial, as it influences healthcare funding, resource allocation, and the broader societal commitment to eradicating TB. This research underscores the importance of integrating clinical efficacy with economic viability to formulate comprehensive TB control strategies that are both effective and sustainable.

## Methods

### Study population

The study considered adults above 18 years and those undergoing treatment for DR-TB in the public health facilities in India. Study population represents a hypothetical cohort of 100,000 patients affected by DR-TB.

### Study perspective

An economic model was conducted using a decision tree analysis to evaluate the cost-effectiveness of the short-course regimens, namely BEAT, BPaL, BPaLM, BPaLC, mBPaL1, mBPaL2, and mBPaL3. Shorter regimens were compared with the current SoC regimen to treat DR-TB. The economic evaluation primarily focused on the health system perspective by considering the costs incurred by it in the NTEP.

### Intervention and comparator

Existing model compared the costs and the outcomes of BEAT, BPaL, BPaLM, BPaLC, mBPaL1, mBPaL2, and mBPaL3 (6–9 months) regimens with the current SoC (9–11 months) regimen for DR-TB patients at public health facilities (Table 1).

**Table 1.** Various regimens to treat DR-TB

Strategy	Drugs	Duration	Regimen
Proposed strategy 1 (BEAT)	Bedaquiline (Bdq) Delamanid (Dlm) Clofazimine (Cfz) Linezolid (Lzd)	6–9 months	(26 weeks) Bdq Dlm Cfz_100_ Lzd_600_
Proposed strategy 2 (BPaL)	Bedaquiline (Bdq) Pretomanid (Pa) Linezolid (Lzd)	6–9 months	(26 weeks) Bdq Pa Lzd
Proposed strategy 3 (BPaLM)	Bedaquiline (Bdq) Pretomanid (Pa) Linezolid (Lzd) Moxifloxacin (Mfx)	6–9 months	(24 weeks) Bdq Pa Lzd Mfx_400_
Proposed strategy 4 (BPaLC)	Bedaquiline (Bdq) Pretomanid (Pa) Linezolid (Lzd) Clofazimine (Cfz)	6–9 months	(24 weeks) Bdq Pa Lzd Cfz
Proposed strategy 5 (mBPaL1)	(Modified) Bedaquiline (Bdq) Pretomanid (Pa) Linezolid (Lzd)	6–9 months	(26 weeks) Bdq Pa Lzd_600_
Proposed strategy 6 (mBPaL2)	(Modified) Bedaquiline (Bdq) Pretomanid (Pa) Linezolid (Lzd)	6–9 months	(9 weeks) Bdq Pa Lzd_600_ + (17 weeks) Bdq Pa Lzd_300_
Proposed strategy 7 (mBPaL3)	(Modified) Bedaquiline (Bdq) Pretomanid (Pa) Linezolid (Lzd)	6–9 months	(13 weeks) Bdq Pa Lzd_600_ + (13 weeks) Bdq Pa Lzd_300_
Current strategy (SoC)	Bedaquiline (Bdq) Livofloxacin (Lfx) Clofazimine (Cfz) Pyrazinamide (Z) Ethambutol (E) Isoniazid (Hh) Ethionamide (Eto)	9–11 months	(4–6 months) Bdq Lfx Cfz Z E Hh Eto

**Intervention:** The BPaL regimen includes Bedaquiline, Pretomanid, and Linezolid. The mBPaL1 regimen consists of Bedaquiline and Pretomanid along with Linezolid with a dosage of 600 mg for 26 weeks. The mBPaL2 regimen adjusts the Linezolid dosage to 300 mg for optimal efficacy, safety, and tolerability at a defined time point of 9 weeks. The mBPaL3 regimen includes Bedaquiline, Pretomanid, and Linezolid with a structured dose reduction planned at 13 weeks. The BPaLM regimen adds Moxifloxacin to the BPaL regimen to enhance its effectiveness. The BPaLC regimen introduces Clofazimine to improve treatment outcomes. The BEAT regimen consists of Bedaquiline, Delamanid, Linezolid, and Clofazamine. The reason for selecting these Bedaquiline-based regimens was that the new controlled clinical trials are being conducted to find out effective shorter duration regimens to treat MDR-TB in India. These regimens considered different combinations of drugs and dosages.

**Comparator:** The current SoC regimen for DR-TB includes Bedaquiline, Levofloxacin/Moxifloxacin, Clofazimine, Ethambutol, Ethionamide, high-dose Isoniazid, and Pyrazinamide. This intensive phase lasts for 4–6 months. It is followed by a continuation phase of 5 months, with Levofloxacin/Moxifloxacin, Clofazimine, Ethambutol, and Pyrazinamide, while Bedaquiline is used for a total of 6 months. This shorter oral Bedaquiline-containing DR-TB regimen of 9- to 11-month duration is recommended in eligible patients with confirmed DR-TB who have not been exposed to treatment with second-line TB medicines used in this regimen for more than 1 month, and in whom resistance to fluoroquinolones has been excluded. Shorter oral Bedaquiline-containing DR-TB regimen was introduced in the country in a phased manner to gain programmatic experience to guide future expansion.

### Time horizon

Incremental costs from the health system perspective and QALYs gained were modeled over the full course of the treatment period of the short-course regimens for 6–9 months and current SoC for 9–11 months. The costs and outcomes of all the regimens for that particular time period were considered.

### Model structure

We used a decision-tree economic model to evaluate the cost-effectiveness of different DR-TB treatment regimens from the healthcare system’s perspective. This study focuses on evaluating the cost and effect of the current SoC regimen with the proposed BEAT, BPaL, BPaLM, BPaLC, mBPaL1, mBPaL2, and mBPaL3 regimens.

### Decision tree

**Figure 1.**
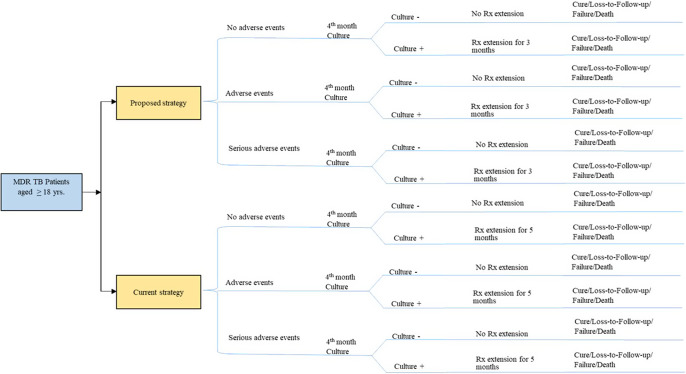
Decision tree for treating DR-TB patients.

**Figure 2.**
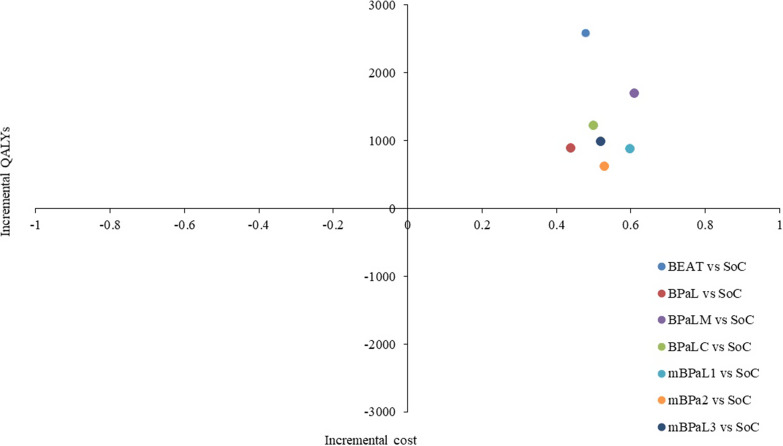
Cost-effectiveness plane.

In Excel (Microsoft, Redmond, WA, USA) and TreeAge Pro (TreeAge Software, LLC, version 2021 R1.2), a deterministic decision tree model for cost-effectiveness analysis was created. We assumed a hypothetical cohort of 100,000 DR-TB patients initiating treatment in public health facilities as the basis for our model. At the start of the model, the cohort was assumed to have a mean age of 32 years based on the average age of TB patients, typical for participants receiving BEAT, BPaL, BPaLM, BPaLC, mBPaL1, mBPaL2, and mBPaL3 regimens. The decision tree (Figure 1) for each strategy branches into adverse drug reactions (ADRs), serious ADRs, and no ADRs. Subsequently, it splits into solid sputum culture test as positive or negative. If the fourth-month culture test is positive in 6- to 9-month regimens, treatment is extended from 6 months to another 3 months; if negative, there is no treatment extension. Similarly, if the fourth-month culture test is positive in 9- to 11-month regimen, treatment is extended from 6 months to another 5 months; if negative, there is no treatment extension. Further branches lead to treatment outcomes, such as cure, loss to follow-up (LTF), treatment failure, and death (Figure 2).

### Model input parameters

The key input parameters used in the model are given in Table 2. It includes the average age of TB patients (23), age-specific life expectancy, and all-cause mortality (24). It also includes TB treatment outcomes, ADR, costs of treatment, and quality of life (QoL) of different regimens.

**Table 2.** Input parameters used for the cost-effectiveness analysis of the 6-month short-course regimens compared to the 9- to 11-month standard of care to treat DR-TB

	Input parameters	Base case	Lower	Upper	Distribution	Source
Demographic values	Average age of TB patients	32	32	32	Normal	23
	Life expectancy at age 32 years	44	44	44	NA	24
	Cohort population	100,000	100,000	100,000	NA	Assumption
Current standard of care (9–11 months)	Cure	0.710	0.570	0.850	Beta	33,34
	LTF	0.110	0.090	0.130	Beta	33,34
	Failure	0.020	0.020	0.020	Beta	33,34
	Death	0.150	0.120	0.180	Beta	33,34
	No ADR	0.200	0.160	0.230	Beta	13
	ADR	0.480	0.390	0.580	Beta	13
	Serious ADR	0.320	0.260	0.390	Beta	13
	Culture positive	0.260	0.210	0.310	Beta	38
BEAT	Cure	0.850	0.680	1.000	Beta	28
	LTF	0.120	0.100	0.140	Beta	28
	Failure	0.010	0.010	0.010	Beta	28
	Death	0.020	0.020	0.020	Beta	28
	No ADR	0.040	0.030	0.050	Beta	28
	ADR	0.810	0.650	0.970	Beta	28
	Serious ADR	0.150	0.120	0.180	Beta	28
	Culture positive	0.150	0.120	0.180	Beta	28
BPaL	Cure	0.900	0.720	1.000	Beta	18,19,29–32
	LTF	0.040	0.030	0.050	Beta	18,19,29–32
	Failure	0.020	0.020	0.020	Beta	18,19,29–32
	Death	0.040	0.030	0.050	Beta	18,19,29–32
	No ADR	0.120	0.100	0.140	Beta	Estimated
	ADR	0.620	0.500	0.740	Beta	29
	Serious ADR	0.260	0.210	0.310	Beta	39
	Culture positive	0.100	0.080	0.120	Beta	38
BPaLM	Cure	0.960	0.760	1.000	Beta	19,29,30
	LTF	0.040	0.030	0.050	Beta	19,29,30
	Failure	0.000	0.000	0.000	Beta	19,29,30
	Death	0.000	0.000	0.000	Beta	19,29,30
	No ADR	0.000	0.000	0.000	Beta	Estimated
	ADR	0.800	0.640	0.960	Beta	29
	Serious ADR	0.200	0.160	0.240	Beta	39
	Culture positive	0.110	0.090	0.130	Beta	38
BPaLC	Cure	0.900	0.720	1.000	Beta	19
	LTF	0.060	0.040	0.070	Beta	19
	Failure	0.020	0.020	0.030	Beta	19
	Death	0.020	0.020	0.030	Beta	19
	No ADR	0.000	0.000	0.000	Beta	Estimated
	ADR	0.610	0.490	0.740	Beta	29
	Serious ADR	0.390	0.310	0.460	Beta	39
	Culture positive	0.160	0.130	0.190	Beta	38
mBPaL1	Cure	0.940	0.760	1.000	Beta	40
	LTF	0.010	0.010	0.010	Beta	40
	Failure	0.050	0.040	0.060	Beta	40
	Death	0.000	0.000	0.000	Beta	40
	No ADR	0.012	0.090	0.140	Beta	40
	ADR	0.820	0.660	0.990	Beta	40
	Serious ADR	0.060	0.050	0.070	Beta	40
	Culture positive	0.050	0.040	0.060	Beta	40
mBPaL2	Cure	0.960	0.770	1.000	Beta	40
	LTF	0.000	0.000	0.000	Beta	40
	Failure	0.020	0.010	0.020	Beta	40
	Death	0.020	0.020	0.030	Beta	40
	No ADR	0.000	0.000	0.000	Beta	40
	ADR	0.900	0.720	1.000	Beta	40
	Serious ADR	0.100	0.080	0.120	Beta	40
	Culture positive	0.040	0.030	0.050	Beta	40
mBPaL3	Cure	0.940	0.750	1.000	Beta	40
	LTF	0.000	0.000	0.000	Beta	40
	Failure	0.030	0.030	0.040	Beta	40
	Death	0.020	0.020	0.030	Beta	40
	No ADR	0.130	0.100	0.150	Beta	40
	ADR	0.810	0.650	0.980	Beta	40
	Serious ADR	0.060	0.050	0.070	Beta	40
	Culture positive	0.040	0.030	0.050	Beta	40
Mortality	ACM	0.010	0.008	0.012	Beta	24
Quality of life	Cure	0.810	0.660	1.000	Beta	36
	LTF	0.700	0.560	0.840	Beta	35
	Failure	0.700	0.560	0.840	Beta	35
Drug cost	SoC 9–11 months	24784	19827	29741	Gamma	25
	BEAT	51777	41422	62132	Gamma	25
	BPaL	37279	29823	44735	Gamma	25
	BPaLM	39738	31790	47686	Gamma	25
	BPaLC	38959	31167	46751	Gamma	25
	mBPaL1	38158	30526	45790	Gamma	25
	mBPaL2	35130	28104	42156	Gamma	25
	mBPaL3	39473	31578	47368	Gamma	25
Treatment extension cost	SoC 9–11 months	23153	18523	27784	Gamma	Estimated
	BEAT	27389	21911	32866	Gamma	Estimated
	BPaL	20140	16112	24167	Gamma	Estimated
	BPaLM	21369	17095	25643	Gamma	Estimated
	BPaLC	20980	16784	25175	Gamma	Estimated
	mBPaL1	20579	16463	24695	Gamma	Estimated
	mBPaL2	19065	15252	22878	Gamma	Estimated
	mBPaL3	21237	16989	25484	Gamma	Estimated
Other treatment cost	Chest X-ray (digital)	64	51	77	Gamma	27
	ECG	1495	1196	1794	Gamma	27
	Nutrition support	500	400	700	Gamma	26
	Treatment honorarium	2000	1600	2400	Gamma	26
	Full hemogram	62	50	74	Gamma	27
	Blood sugar	76	61	91	Gamma	27
	Thyroid-stimulating hormone test	260	208	312	Gamma	27
	Liver function test	260	208	312	Gamma	27
	Hospitalization of ADR	4945	3956	5934	Gamma	27
	Sputum culture test	184	147	221	Gamma	27
WTP	Willingness-to-pay threshold	124600	124600	124600	NA	41

#### Cost data

We estimated the healthcare system’s costs of DR-TB treatment by combining DR-TB treatment guidelines, previously published price estimates, and information from the Central TB Division, Ministry of Health and Family Welfare, Government of India. The costs for pretreatment investigation were obtained from published literature. The costs for Bedaquiline, Pretamonid, and Linezolid drugs were given by Central TB Division (25). Treatment extension cost is calculated based on the treatment extension by months using drug cost. Information on nutrition support and treatment honorarium was collected from Nikshay Poshan Yojana, Central TB Division (26). The total cost for nutrition support was calculated based on the duration of treatment using per-month nutrition cost. Costs for chest X-ray (digital), electrocardiogram, full hemogram, blood sugar, thyroid-stimulating hormone test, liver function test, hospitalization of ADRs, and sputum culture test were collected from the study done in India (27).

#### Effectiveness data

The clinical outcomes of the BEAT (28) and BPaLC (29) regimens were collected from the Indian Council of Medical Research - National Institute for Research in Tuberculosis (ICMR-NIRT) study and 24-week all-oral regimen for rifampin-resistant TB literature, respectively, whereas the treatment outcomes of BPaL (18;19;29–32), BPaLM (19;29;30), and current SoC 9–11 months (33,34) were estimated by meta-analysis. A random-effects model was used to create pooled summary estimates of treatment outcomes for BPaL (from six studies), BPaLM (from three studies), and the current SoC (9–11 months, from two studies). Forest plots with 95 percent confidence intervals (CIs) were used to visually evaluate heterogeneity. The QoL score for cured patients was collected from the study conducted among TB patients treated under NTEP in rural and urban Puducherry (35). The QoL scores of LTF and failure were collected from the study done among TB patients in Malawi (36). QoL scores were considered the same for each treatment outcome irrespective of the regimen. The assumption was made based on a systematic review that reported almost similar QoL scores on the completion of TB treatment, ranging from 0.88 to 0.91 with a standard deviation of 0.11–0.32 (37). Data for sputum culture conversion at the fourth month, ADRs, and serious ADRs for the BEAT were collected from the ICMR-NIRT study (28), and data for BPaL, BPaLM, BPaLC, and SoC 9- to 11-month regimen were collected and estimated by meta-analysis (38).ADRs and serious ADRs for BPaL, BPaLM, and BPaLC were also collected from the multicenter randomized controlled non-inferiority trial on all oral regimens for DR-TB treatment (29;39). The clinical outcomes, ADRs, and culture conversion at the fourth month for the mBPaL1, mBPaL2, and mBPaL3 regimens were collected from the clinical trial done at ICMR-NIRT (40).

### Model outcome parameters

The model outcomes are denoted in terms of QALYs, life years (LYs), and the total costs incurred for all the regimens. This economic model made a comparison of both incremental costs and incremental effects, and the ICERs were calculated.

### Cost-effectiveness analysis

Cost-effectiveness of short-course regimens was assessed by calculating the ICER between the proposed strategies and the current strategy. The difference in costs and QALYs of the proposed strategies and the current strategy was used to calculate ICER using the following formula:

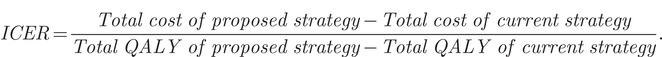



Comparing the ICER with the willingness-to-pay (WTP) thresholds value helps us to conclude whether the intervention is cost-effective. WTP is determined by one-time gross domestic product (GDP) per capita of the country.

### Scenario analysis

We performed different scenario analysis by changing the SoC (9–11 months) to mixed SoC with 90 percent to 42 percent of shorter SoC regimen (9–11 months) and 10 percent to 58 percent of longer SoC regimen (18–20 months). In India, under NTEP, all notified MDR/RR-TB patients were undergoing treatment with the existing mixed SoC regimen in which 58 percent of patients received longer regimen (18–20 months) and 42 percent were on shorter regimen (9–11 months). If a 6 6-month BPaL-based regimen is introduced, it is assumed that the maximum number of people will be eligible for this regimen. Based on this assumption, we have taken the varying percentages of patients on short versus long treatments as 90 percent short and 10 percent long. These proportions constituting mixed SoC were compared with all the proposed regimens, and ICER values were calculated.

### Sensitivity analysis

The model robustness was assessed using sensitivity analysis by changing the input parameters between 20 percent above and below the base case values. One-way sensitivity analysis (OWSA) was used to find out which parameter has greater impact on the ICER value. The uncertainty in outcome variables and their effect on ICER was illustrated in a tornado diagram. Probabilistic sensitivity analysis (PSA) using 1,000 iterations of Monte Carlo simulations with 95 percent CIs was used to validate the model using Microsoft Excel. The 1,000 iterations range was considered, as many published studies and guidelines often use this range, which is reasonable and gives stable estimates without excessive computation. The resulting ICER values were plotted in a scatter plot. The cost-effectiveness acceptability curve (CEAC) was drawn to indicate the model’s probabilistic response to different cost-effectiveness thresholds.

### Willingness to pay

The WTP threshold value used to represent an estimate of what a consumer of health care might be prepared to pay for the health benefit and is often based on a country’s per capita GDP. A one-time GDP per capita of ₹1,24,600 ($1,496) for the year 2023–24 was considered as the WTP threshold and used to compare the threshold to determine the cost-effectiveness of the proposed regimens (41). In health economic decision modeling, GDP per capita is commonly used as the WTP threshold, and the same approach was adopted in this study.

## Results

### Base-case analysis

The base-case analysis for the hypothetical cohort of 100,000 DR-TB patients showed that the total costs incurred by health system for the BEAT, BPaL, BPaLM, BPaLC, mBPaL1, mBPaL2, mBPaL3, and the current SoC regimens were ₹6,804, ₹5,105, ₹5,444, ₹5,436, ₹5,096, ₹4,833, ₹5,204, and ₹4,219 million, respectively. In terms of effectiveness, the total QALYs of BPaLM regimen were higher than those for mBPaL1, followed by mBPaL2, mBPaL3, BPaLC, BEAT, and BPaL, and the current SoCs were 3.50, 3.46, 3.63, 3.52, 3.62, 3.55, 3.54, and 3.02 million, respectively (Table 3). The incremental costs and QALYs of the different DR-TB regimens were compared with their additional expenses and health benefits against the current SoC regimen. All shorter regimens were more cost-effective and more effective compared to the current SoC regimen. Based on the threshold value, these are the regimens that are cost-effective as compared to the current SoC regimen.

**Table 3.** Base-case analysis for all short-course regimens compared with the current standard of care regimen

Strategy	Total (in million)		Incremental (in million)		ICER
	Cost (₹)	QALY	Cost (₹)	QALY	Cost/QALY
BEAT	6,804	3.50	2585	0.48	5,385
BPaL	5,105	3.46	886	0.44	2,014
BPaLM	5,444	3.63	1225	0.61	2,008
BPaLC	5,436	3.52	1217	0.50	2,435
mBPaL1	5,096	3.62	877	0.60	1,462
mBPaL2	4,833	3.55	614	0.53	1,159
mBPaL3	5,204	3.54	985	0.52	1,895
Current SoC	4,219	3.02	–	–	–

### Incremental cost-effectiveness ratio

The ICERs were calculated using incremental costs and incremental QALYs. When all the short-course regimens were compared with the current SoC regimen, the ICER was ₹5385, ₹2014, ₹2008, ₹2435, ₹1462, ₹1159, and ₹1895 for BEAT, BPaL, BPaLM, BPaLC, mBPaL1, mBPaL2, and mBPaL3, respectively. Indicating that, to gain an additional QALY, there is a need to spend ₹5,385, ₹2,014, ₹2,008, ₹2,435, ₹1,462, ₹1,159, and ₹1,895 more for BEAT, BPaL, BPaLM, BPaLC, mBPaL1, mBPaL2, and mBPaL3, respectively. Among the short-course regimens, mBPaL2 is the dominant strategy (a strategy is considered dominant if it is both less costly and more effective than another strategy; it improves health outcomes while also saving money) and mBPaL1 has extended dominance (a situation where a strategy is not eliminated by a single, superior option, but rather by a combination or “mixed strategy” of other options. This means that a strategy may have a higher ICER than a more effective intervention, leading to the elimination of the less effective strategy, even if it is less expensive).

### One-way sensitivity analysis

OWSA was performed and ICERs were plotted in a tornado diagram. For BEAT regimen, the drug cost of BEAT, the QoL of cured patients, and the probability of death in current SoC were the major factors influencing the ICER value (Supplementary Figure S1). For BPaL regimen, the drug cost of BPaL and drug cost of the current SoC influenced the ICER value (Supplementary Figure S2). For BPaLM regimen, the drug cost for BPaLM, the drug cost for current SoC, and the QoL of cured patients influenced the ICER (Supplementary Figure S3). For BPaLC regimen, the drug cost for BPaLC, the drug cost for current SoC, and the QoL of cured patients influenced the ICER (Supplementary Figure S4). For mBPaL1 regimen, the drug cost for mBPaL1, the drug cost for current SoC, and the QoL of cured patients influenced the ICER (Supplementary Figure S5). For mBPaL2 regimen, the drug cost for mBPaL1, the drug cost for current SoC, and the QoL of cured patients influenced the ICER (Supplementary Figure S6). For mBPaL1 regimen, the drug cost for mBPaL1, the drug cost for current SoC, and the QoL of cured patients influenced the ICER (Supplementary Figure S7). Overall, the drug cost is the major influencing factor for all the short-course regimens in determining cost-effectiveness, which in turn was highly dependent on drug pricing.

### Probability sensitivity analysis

Joint cost-effectiveness analysis with PSA was conducted for all shorter regimens compared to the current SoC regimen. For BEAT, PSA showed that the ICER distribution placed 100 percent of iterations in the northeast quadrant, indicating dominance over the current SoC, which is a more cost-effective and more effective regimen (Supplementary Figure S8). Similarly, BPaL, BPaLM, BPaLC, mBPaL1, mBPaL2, and mBPaL3 PSA revealed that 98, 99.5, 97.8, 97.4, 94, and 97 percent of simulations were in the northeast quadrant, respectively, indicating that these regimens were more cost-effective and more effective (Supplementary Figures S9–S14).

### Cost-effectiveness acceptability curve

The CEAC curve indicated that the implementation of different short-course regimens for DR-TB patients had varying probabilities of being economically dominant strategies compared to the current SoC. It showed that all shorter regimens were 100 percent cost-effective, which means our ICER values are less than the WTP threshold (Supplementary Figures S15–S21).

### Scenario analysis

We performed various scenario analyses by changing the proportions of patients put on treatment from the ratio of 10 percent in longer regimen and 90 percent in shorter regimen to 50 percent in longer regimen and 50 percent in shorter regimen. The ICER values for the different proportions of mixed SoC were calculated and presented in Supplementary Table S1. The ICERs for the proportion of mixed SoC of 58 percent longer and 42 percent shorter were compared with BEAT, BPaL, BPaLM, BPaLC, mBPaL1, mBPaL2, and mBPaL3. These values were ₹3,401, ₹76, ₹1,026, ₹632, ₹43, ₹-438, and ₹245, respectively. Further, the ICER for the proportion of mixed SoC of 10 percent longer and 90 percent shorter compared with BEAT, BPaL, BPaLM, BPaLC, mBPaL1, mBPaL2, and mBPaL3. The ICER values were ₹4,976, ₹1,680, ₹1,844, ₹2,099, ₹1,218, ₹849, and ₹1,582, respectively. When the proportion of mixed SoC is changed to 50 percent longer and 50 percent shorter, the estimated ICER for BEAT, BPaL, BPaLM, BPaLC, mBPaL1, mBPaL2, and mBPaL3 were found to be ₹3,631, ₹332, ₹1,152, ₹846, ₹233, ₹-228, and ₹459, respectively. As the proportion of longer regimen in mixed SoC is increased from 50 percent and beyond, ICER for mBPaL2 regimen turned cost-saving, while that of the other six shorter regimens decreased.

## Discussion

This is the first time that we are investigating the cost-effectiveness of seven new shorter, all oral regimens for DR-TB treatment, comparing them to the current SoC regimen in India. We found that all shorter regimens, namely BEAT, BPaL, BPaLM, BPaLC, mBPaL1, mBPaL2, and mBPaL3 regimens were cost-effective with the ICER of ₹5,385, ₹2,014, ₹2,008, ₹2,435, ₹1,462, ₹1,159, and ₹1895, respectively, indicating the additional cost incurred by each regimen to gain an additional QALY. These costs were 14 percent to 61 percent more than the cost of current SoC, which is less than the WTP threshold (one time GDP per capita) of India, indicating the cost-effectiveness. The current study findings highlight that treating DR-TB with Bedaquiline-based shorter regimens is cost-effective for the health system. Similarly, a study conducted on cost-effectiveness of Bedaquiline-based regimens, namely BPaL, BPaLM, and BPaLC, compared with the mix of longer and shorter SoC regimens to treat DR-TB from the health system perspective in India, reported that all three regimens were cost-saving in terms of disability-adjusted LYs (DALYs) averted (42). The estimated cost per DALY averted by BPaL, BPaLM, and BPaLC regimens in India was $-112, $-80, and $-27, respectively (42).

Modeling studies on assessing cost-effectiveness of comparing longer SoC regimen with 6-month BPaL shorter regimen in various countries, such as Philippines, South Africa, Georgia, Pakistan, Ukraine, Belarus, Uzbekistan, Indonesia, Kyrgyzstan, Nigeria, and Moldova, reported that 6-month BPaL shorter regimen is better than the 18- to 20-month longer SoC regimen (42–46). It was reported that the incremental cost-effectiveness of BPaL, BPaLM, and BPaLC compared with the current mix of long and short SoC to treat DR-TB from the health system perspective would improve health outcomes and reduce costs compared with the current programmatic mix of long and short SoC regimens in India, Georgia, Philippines, and South Africa. Based on these scientific successes, the WHO has recommended for programmatic use of BPaL-based regimens, and countries are starting to consider the potential advantages and costs of replacing the current SoC for DR-TB. Subsequently, there have been substantial changes in the landscape of treatment options available for people with DR-TB. In the current study, we compared 9- to 11-month shorter SoC regimen with 6-month Bedaquiline-based regimens. We found that 6-month Bedaquiline-based regimens are cost-effective as compared to 9- to 11-month shorter SoC regimens. The SoC regimen often requires long treatment durations ranging from 9 to 20 months, involving many drugs with potential side effects, and has lower cure rates (43). Long-term treatment and a high pill burden can have a strong negative impact on the patient’s QoL (19). The new regimens, although initially with higher costs, provide promising results in terms of 3 months reduction in treatment period, better adherence rates with 7 percent less LTF, 6 percent less serious ADR, and 20 percent improvement in cure rates (44–46). It was also estimated that an increased number of patients would be treated successfully with Bedaquiline-based regimens due to the shorter duration (47). It resulted in saving many lives and averting DALYs per annum (47). These advantages highlight its potential for substantial long-term savings, both in terms of healthcare costs and broader societal benefits. It was reported by Sweeney et al. that BPaL regimen was the most cost-saving by $112–$1,173 per person in the Philippines, South Africa, Georgia, and India. It was recommended that countries should consider programmatic uptake of BPaL-based regimens. The cost-effectiveness of our study might be from the underestimation of benefits of shorter Bedaquiline-based regimens, such as reduction in hospitalization, lower failure, relapse, and lower transmission rate. If we had considered these factors, the effect size would have been higher.

The other dimension is that the Bedaquiline-based regimens were also compared in various aspects of longer duration of SoC and XDR-TB. In this aspect, the four-country model on introduction of BPaL and BPaLM regimens at the country level found that its implementation resulted in higher savings per year in Pakistan, Philippines, South Africa, and Ukraine at about $2.6, $3.8, $6, $9 million, respectively (42). The reason for cost-saving could be due to the comparison of 18- to 20-month SoC regimen, while the current study is comparing 6- to 9-month regimen. When we compared the 6-month Bedaquiline-based regimens with longer SoC, it was the dominant strategy. Among short-course regimens, when compared with shorter SoC, mBPaL2 was the dominant strategy, and mBPaL1 became an extended dominance strategy. It was reported that the increased benefits of shorter SoC are due to the shortening of the regimen and the better treatment outcomes (47). In addition, the budgetary impact of introducing BPaL in Indonesia, Kyrgyzstan, and Nigeria reported BPaL regimen would make an average reduction of 15 percent to 32 percent in budgets required to manage patients with XDR-TB in the respective countries (45).

The economic evaluation of the Bedaquiline-based regimens was studied by analyzing different health outcomes and calculating average cost-effectiveness ratio (ACER) or ICER per successful treatment outcome, DALY averted, and QALY gained. A study on cost-effectiveness of comparing 9- to 11-month SoC with BPaL regimen in the Philippines estimated that the ACER was $1,994.5 and ICER was $-469 per successful treatment (48). Another study on cost-effectiveness of BPaL was compared with the 18-month SoC regimen in South Africa, Georgia, and the Philippines. It was reported that ICER per DALY was $-165, $-406, and $-387, respectively, indicating BPaL regimen is more likely to be cost-saving in all study settings (44). The magnitude of these savings depends on the prevalence of DR-TB in the country. It was estimated that savings for different countries ~$3 million for South Africa, $200,000 for Georgia, and $60,000 for the Philippines (44). Another study estimated that the cost-effectiveness of BPaL, BPaLM, and BPaLC regimens compared with the mix of longer and shorter SoC regimens to treat DR-TB from the health system perspective, the savings were $112–$1,173 per person (42). A mathematical modeling study on cost-effectiveness of the 6-month BPaLM regimen compared with 9- to 18-month SoC regimens for DR-TB in Moldova found that the 6-month BPaLM regimen is cost-effective with substantial reductions in the duration and cost of treatment (49). Further, it was also reported that compared to the SoC, BPaLM will reduce lifetime costs by $3,366 per individual. It was recommended that the implementation of the 6-month BPaLM regimen could improve the cost-effectiveness of care for individuals diagnosed with DR-TB, particularly in settings with a high burden of MDR-TB (45).

The other important finding from OWSA was that, of all the health systems cost-effectiveness components, BPaL drug costs and QoL score of cured patients constituted the largest contributor to the overall cost-saving. If the cost of BPaL regimen is reduced 20 percent, the ICER will decrease by 87 percent and become a very cost-effective strategy. Currently, Bedaquiline drug is patented; if the patent is removed, the cost will reduce and the regimen might turn cost-saving. It was reported that reducing the Bedaquiline price from $1.81 to $1.00 per tablet made the regimen cost-effective from the health system perspective in India and Moldova (50). While the cost of implementing the Bedaquiline-based regimens may initially appear high, if countries implemented this regimen through negotiated procurement by bulk buying, this will become economically more viable. In addition, the current analysis is restricted to a health system perspective. If we considered costs incurred by patients and their households, this would be more beneficial from a societal perspective. We may have underestimated the benefits of this regimen to society. Since this is the first time we estimated the economic efficiency of these regimens, there is a need for further studies on the real-world scenario of Bedaquiline-based regimens.

## Limitations of the study

In the current study, we considered only the health system perspective; this may underestimate the benefits of shorter Bedaquiline-based regimens. Further, we have interpreted the results with a few limitations. First one being the fact that we did not consider the treatment outcomes categorized by different ADR and treatment outcomes after culture conversion. Second is that the ADR of Linezolid are comparatively higher than those of other drugs, which we have not accounted for in our model. Third is that we have not fully accounted for the benefits of shorter Bedaquiline-based regimens, such as reduction in hospitalization, lower failure, relapse, and lower transmission rates. Fourth is that due to nonavailability of follow-up data on long-term outcomes like relapse and death after treatment completion, we could not use Markov modeling in the current study. Further, we did not consider other benefits of shorter Bedaquiline-based regimens, such as reducing hospitalization, lower failure, relapse, less transmission, and reducing out-of-pocket expenditure. Still, it is an underestimation of the benefits of shorter Bedaquiline-based regimens.

## Conclusion

This study shows that all short-course Bedaquiline-based regimens are more cost-effective than the current SoC regimen. The implementation of Bedaquiline-based regimen to treat DR-TB in India has become more effective, shorter, and less burdensome to the health system. Overall, the drug cost is a crucial factor for all the short-course regimens in determining cost-effectiveness. Since the Government of India has announced BPaL regimen to be implemented to treat DR-TB, this will provide evidence to make a scientifically evidence-based policy. This benefit may likely increase when the government scales up this intervention with the TB control program.

## Supporting information

Muniyandi et al. supplementary material 1 Muniyandi et al. supplementary material

Muniyandi et al. supplementary material 2 Muniyandi et al. supplementary material

## Data Availability

The article contains the actual research findings, and further inquiries can be directed to the corresponding author.

## Supplementary material

The supplementary material for this article can be found at http://doi.org/10.1017/S0266462325100329.
