# Supplementary material for: Comparing cost-effectiveness of short-course regimens for drug-resistant tuberculosis treatment in India
Source: Int J Technol Assess Health Care. 2025 Jul 21;41(1):e48. doi: 10.1017/S0266462325100329 (PMC12322854; doi:10.1017/S0266462325100329)
Supplement: Muniyandi et al. supplementary material 1 — Muniyandi et al. supplementary material [file S0266462325100329sup001.docx]

**Comparing Cost-Effectiveness of Short Course Regimens for Drug-Resistant Tuberculosis Treatment in India**

Malaisamy Muniyandi^1^, Balaji Ramraj^2^, Sathishkumar Vadamalai^1^, Sahil Abdul Salam^1^, Bella Devaleenal^2^, Jyoti Jaju^3^ and Chandrasekaran Padmapriyadarsini^2^

^1^ Department of Health Economics, ICMR-National Institute for Research in Tuberculosis, Chennai, India

^2^ Department of Clinical Research, ICMR-National Institute for Research in Tuberculosis, Chennai, India

^3^ iDFFEAT TB Project, International Union Against Tuberculosis and Lung Disease, New Delhi, India

**Supplementary Tables and Figures**

**Table S1.** Estimated ICER values of different proportion of mixed standard of care

| Duration | Long | Short | Long | Short | Long | Short | Long | Short | Long | Short | Long | Short |
| --- | --- | --- | --- | --- | --- | --- | --- | --- | --- | --- | --- | --- |
| Proportion | 58% | 42% | 10% | 90% | 20% | 80% | 30% | 70% | 40% | 60% | 50% | 50% |
| BEAT | 3401 | | 4976 | | 4677 | | 4290 | | 3997 | | 3631 | |
| BPaL | 76 | | 1680 | | 1317 | | 991 | | 651 | | 332 | |
| BPaLM | 1026 | | 1844 | | 1681 | | 1493 | | 1332 | | 1152 | |
| BPaLC | 692 | | 2099 | | 1811 | | 1495 | | 1213 | | 846 | |
| mBPaL1 | 43 | | 1218 | | 958 | | 717 | | 469 | | 233 | |
| mBPaL2 | -438 | | 849 | | 578 | | 301 | | 34 | | -228 | |
| mBPaL3 | 245 | | 1582 | | 1306 | | 1010 | | 739 | | 459 | |

**Table S2.** CHEERS checklist—Items to include when reporting economic evaluations of health interventions

| **Section/item** | **Item No** | **Recommendation** | **Reported on page No/ line No** |
| --- | --- | --- | --- |
| **Title** | | | |
|  | 1 | Identify the study as an economic evaluation and specify the interventions being compared. | Page 1 |
| **Abstract** | 2 | Provide a structured summary that highlights context, key methods, results, and alternative analyses. | Page 2 |
| **Introduction** | | | |
| **Background and objectives** | 3 | Give the context for the study, the study question, and its practical relevance for decision making in policy or practice. | Page 3-4 |
| **Methods** | | | |
| **Health economic analysis plan** | 4 | Indicate whether a health economic analysis plan was developed and where available. | - |
| **Study population** | 5 | Describe characteristics of the study population (such as age range, demographics, socioeconomic, or clinical characteristics). | Page 4 |
| **Setting and location** | 6 | Provide relevant contextual information that may influence findings. | Page 4 |
| **Comparators** | 7 | Describe the interventions or strategies being compared and why chosen. | Page 5 |
| **Perspective** | 8 | State the perspective(s) adopted by the study and why chosen. | Page 4 |
| **Time horizon** | 9 | State the time horizon for the study and why appropriate. | Page 5 |
| **Discount rate** | 10 | Report the discount rate(s) and reason chosen. | - |
| **Selection of outcomes** | 11 | Describe what outcomes were used as the measure(s) of benefit(s) and harm(s). | Page 7 |
| **Measurement of outcomes** | 12 | Describe how outcomes used to capture benefit(s) and harm(s) were measured. | Page 7 |
| **Valuation of outcomes** | 13 | Describe the population and methods used to measure and value outcomes. | Page 6-7 |
| **Measurement and valuation of resources and costs** | 14 | Describe how costs were valued. | Page 6 |
| **Currency, price date, and conversion** | 15 | Report the dates of the estimated resource quantities and unit costs, plus the currency and year of conversion. | - |
| **Rationale and description of model** | 16 | If modelling is used, describe in detail and why used. Report if the model is publicly available and where it can be accessed. | Page 6 |
| **Analytics and assumptions** | 17 | Describe any methods for analysing or statistically transforming data, any extrapolation methods, and approaches for validating any model used. | - |
| **Characterising heterogeneity** | 18 | Describe any methods used for estimating how the results of the study vary for subgroups. | - |
| **Characterising distributional effects** | 19 | Describe how impacts are distributed across different individuals or adjustments made to reflect priority populations. | - |
| **Characterising uncertainty** | 20 | Describe methods to characterise any sources of uncertainty in the analysis. | Page 7-8 |
| **Approach to engagement with patients and others affected by the study** | 21 | Describe any approaches to engage patients or service recipients, the general public, communities, or stakeholders (such as clinicians or payers) in the design of the study. | - |
| **Results** | | | |
| **Study parameters** | 22 | Report all analytic inputs (such as values, ranges, references) including uncertainty or distributional assumptions. | Table 2 |
| **Summary of main results** | 23 | Report the mean values for the main categories of costs and outcomes of interest and summarise them in the most appropriate overall measure. | Page 8-9 & Table 3 |
| **Effect of uncertainty** | 24 | Describe how uncertainty about analytic judgments, inputs, or projections affect findings. Report the effect of choice of discount rate and time horizon, if applicable. | Page 9 & Figure S1-S21 |
| **Effect of engagement with patients and others affected by the study** | 25 | Report on any difference patient/service recipient, general public, community, or stakeholder involvement made to the approach or findings of the study | - |
| **Discussion** | | | |
| **Study findings, limitations, generalisability, and current knowledge** | 26 | Report key findings, limitations, ethical or equity considerations not captured, and how these could affect patients, policy, or practice. | Page 10-12 |
| **Other relevant information** | | | |
| **Source of funding** | 27 | Describe how the study was funded and any role of the funder in the identification, design, conduct, and reporting of the analysis | Page 13 |
| **Conflicts of interest** | 28 | Report authors conflicts of interest according to journal or International Committee of Medical Journal Editors requirements. | Page 13 |

For consistency, the CHEERS statement checklist format is based on the format of the CONSORT statement checklist

**Figure S1.** Tornado Diagram for BEAT with current SoC


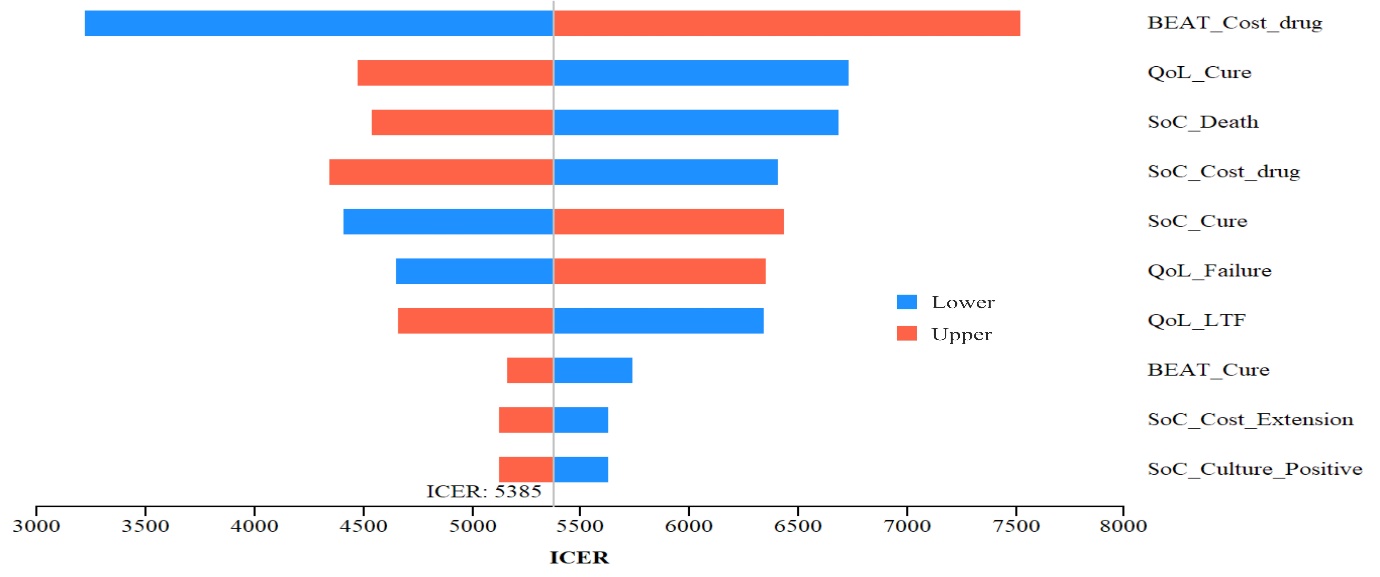


**
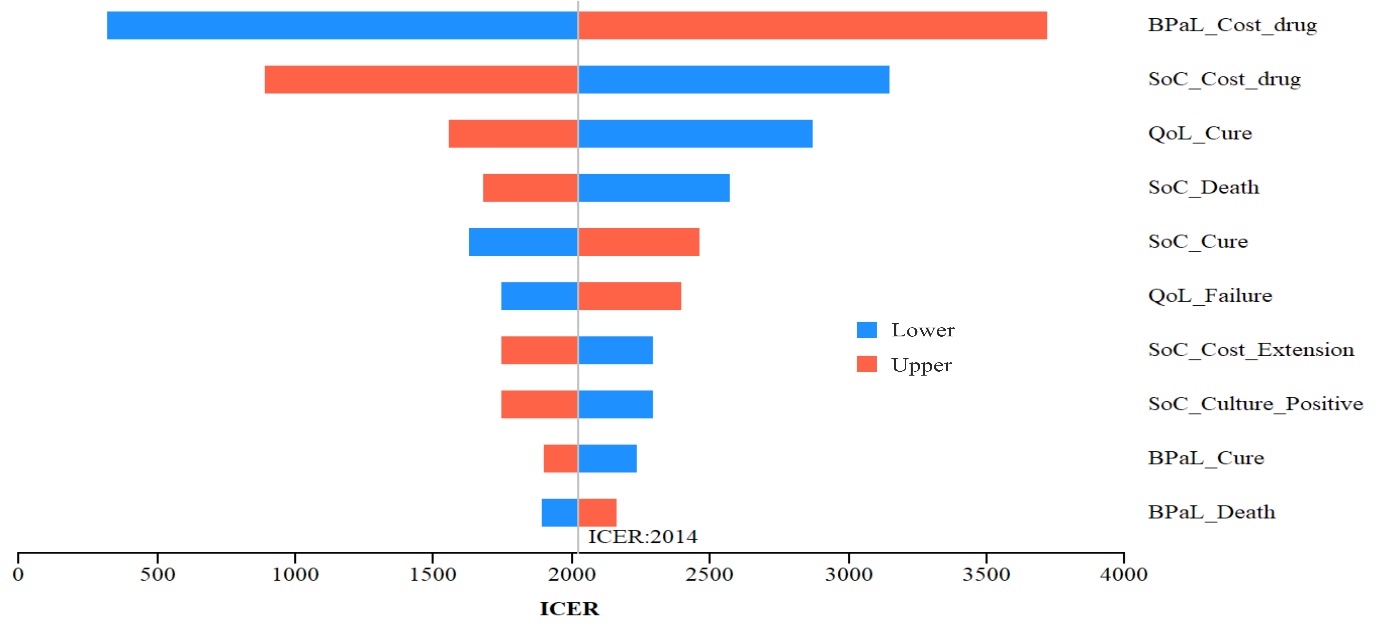
Figure S2.** Tornado Diagram for BPaL with current SoC

**Figure S3.** Tornado Diagram for BPaLM with current SoC


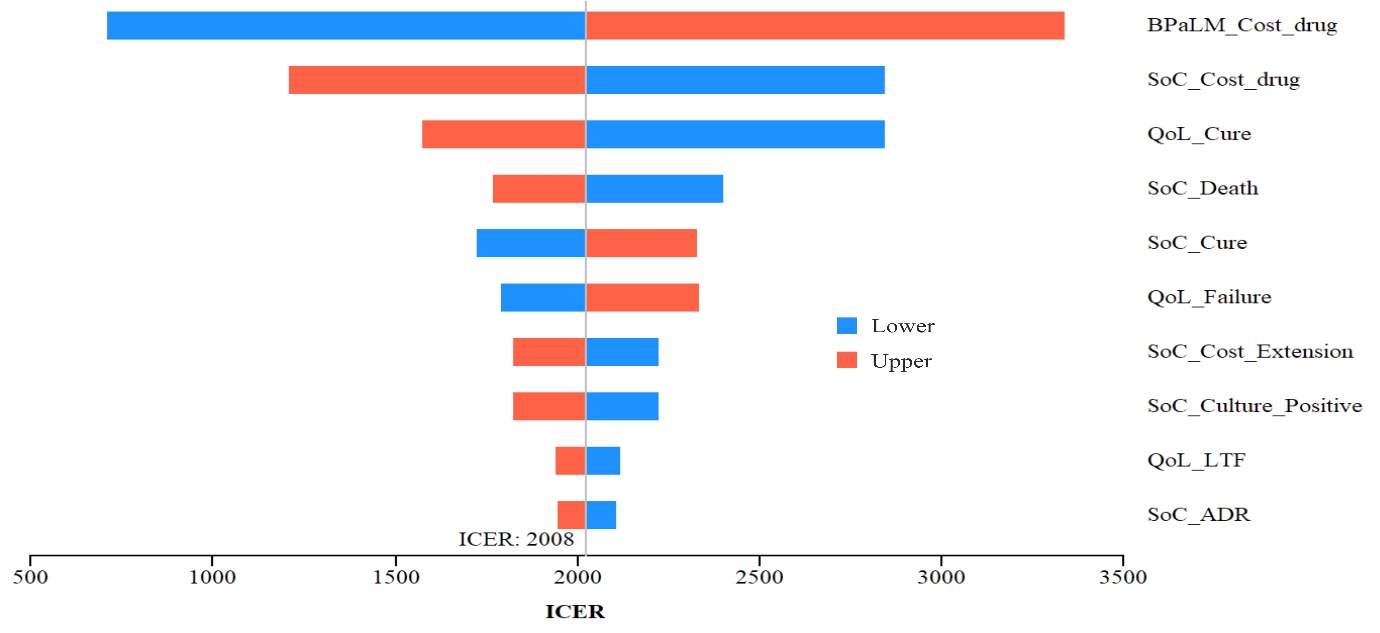


**
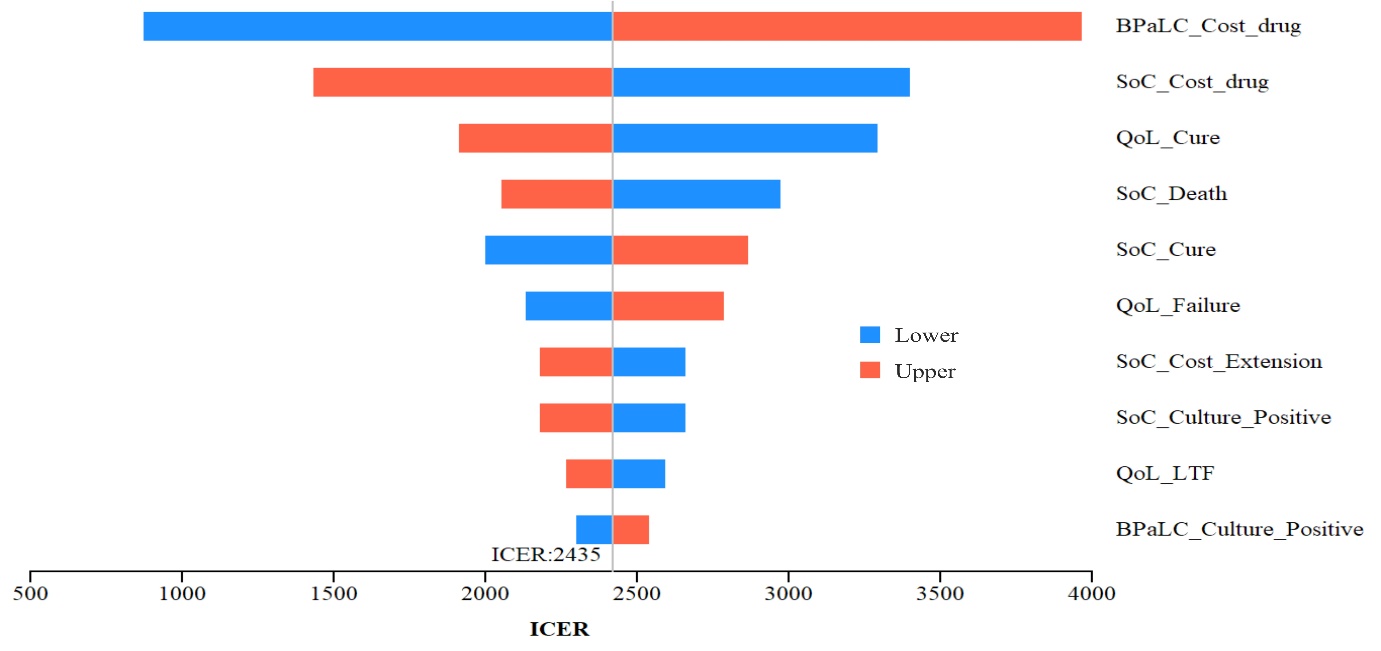
Figure S4.** Tornado Diagram for BPaLC with current SoC


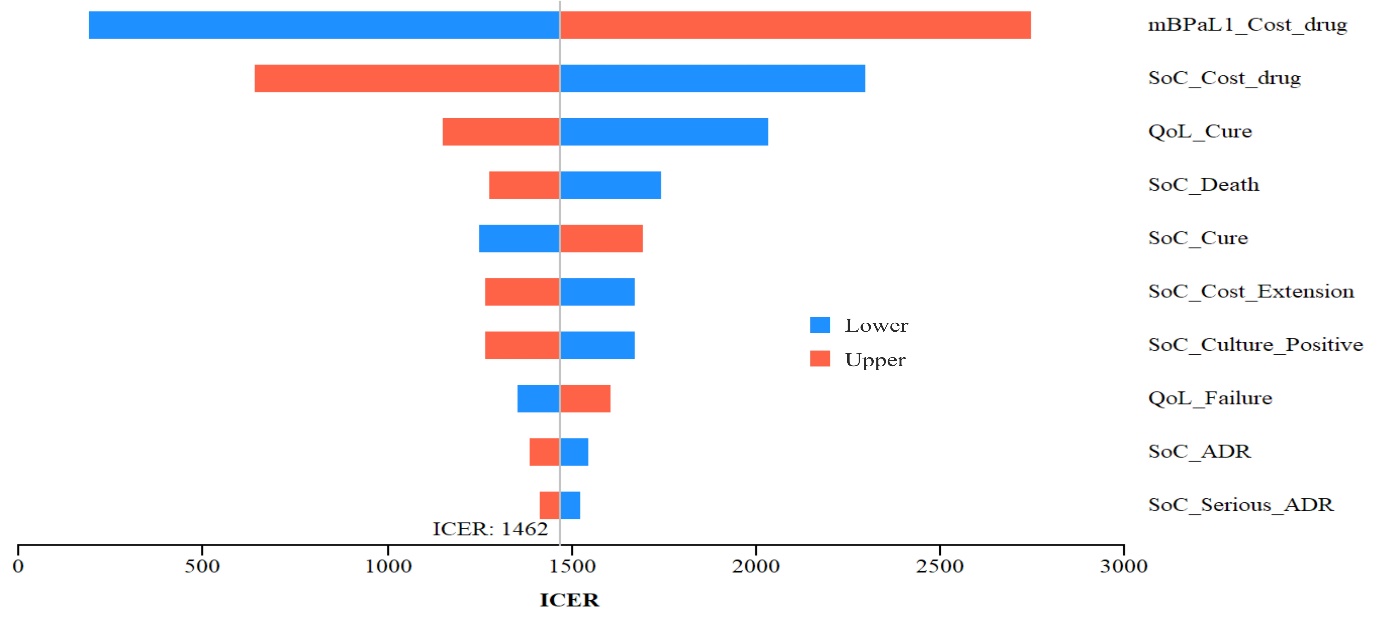
**Figure S5.** Tornado Diagram for mBPaL1 with current SoC

**
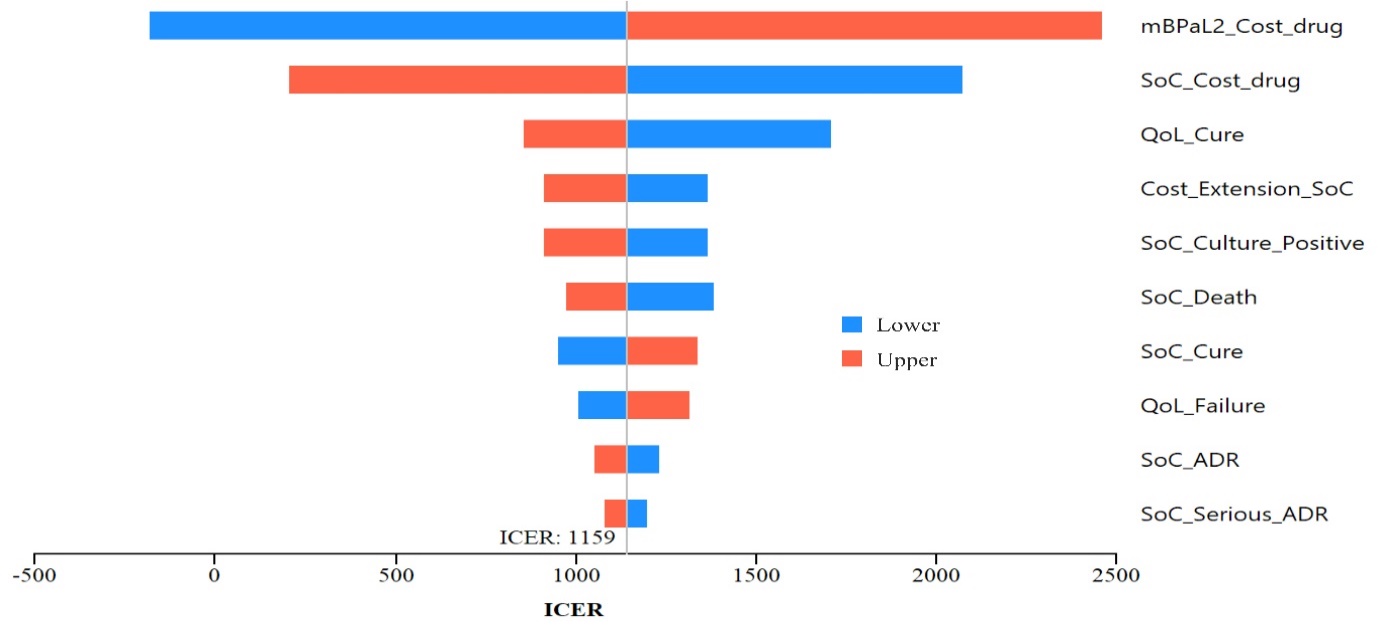
Figure S6.** Tornado Diagram for mBPaL2 with current SoC

**
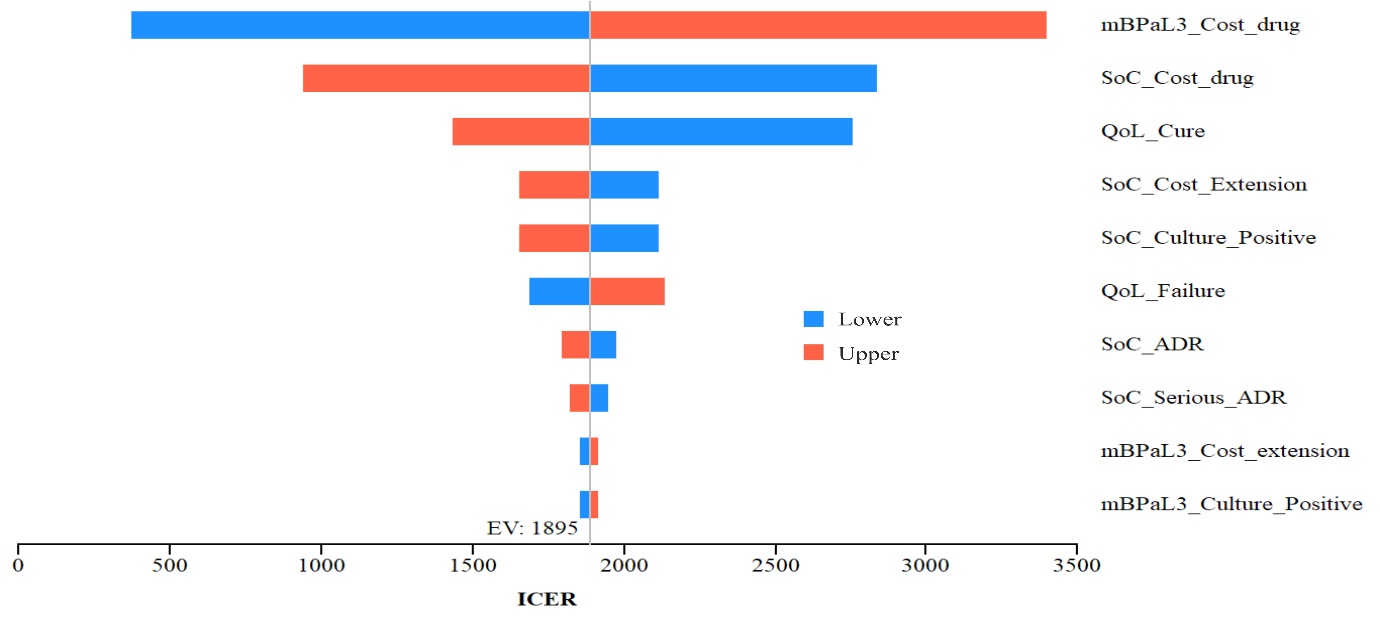
Figure S7.** Tornado Diagram for mBPaL3 with current SoC

**
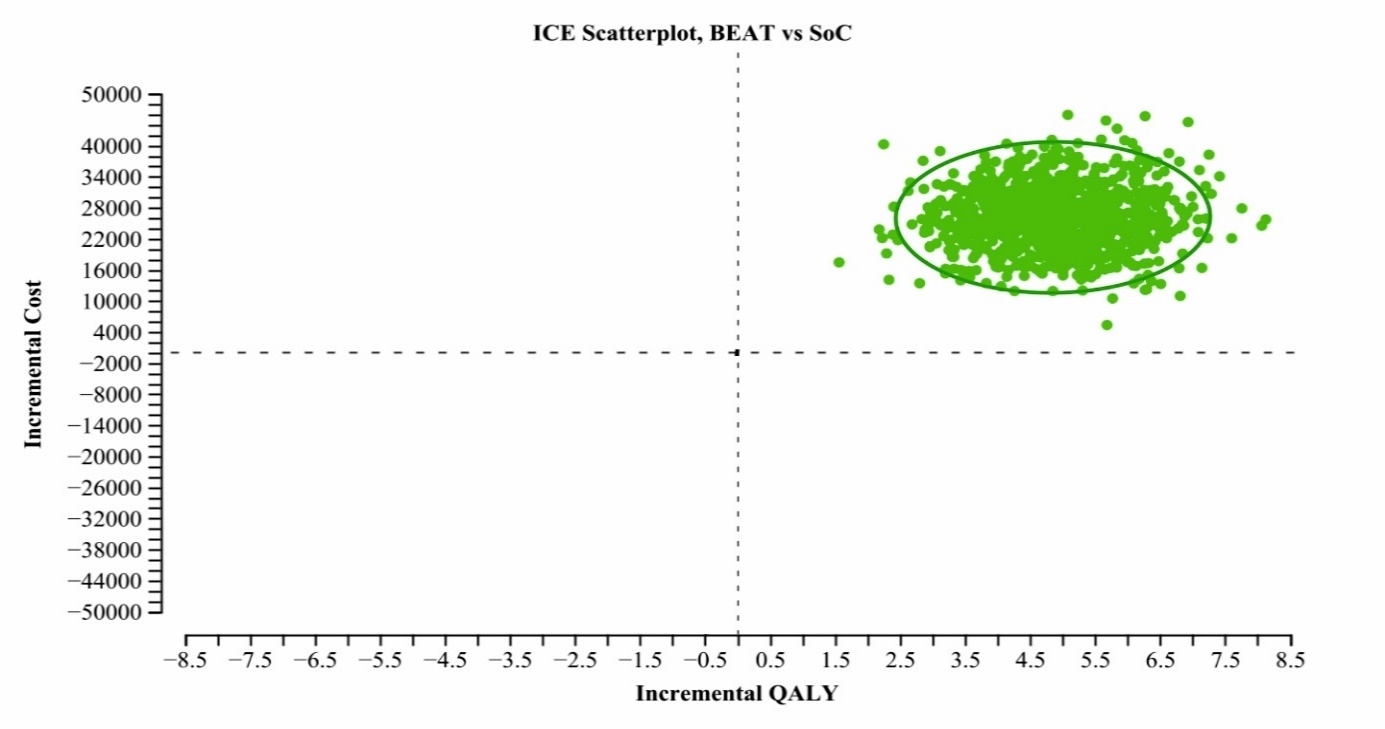
Figure S8.** BEAT with current SoC PSA joint incremental cost and QALY

**
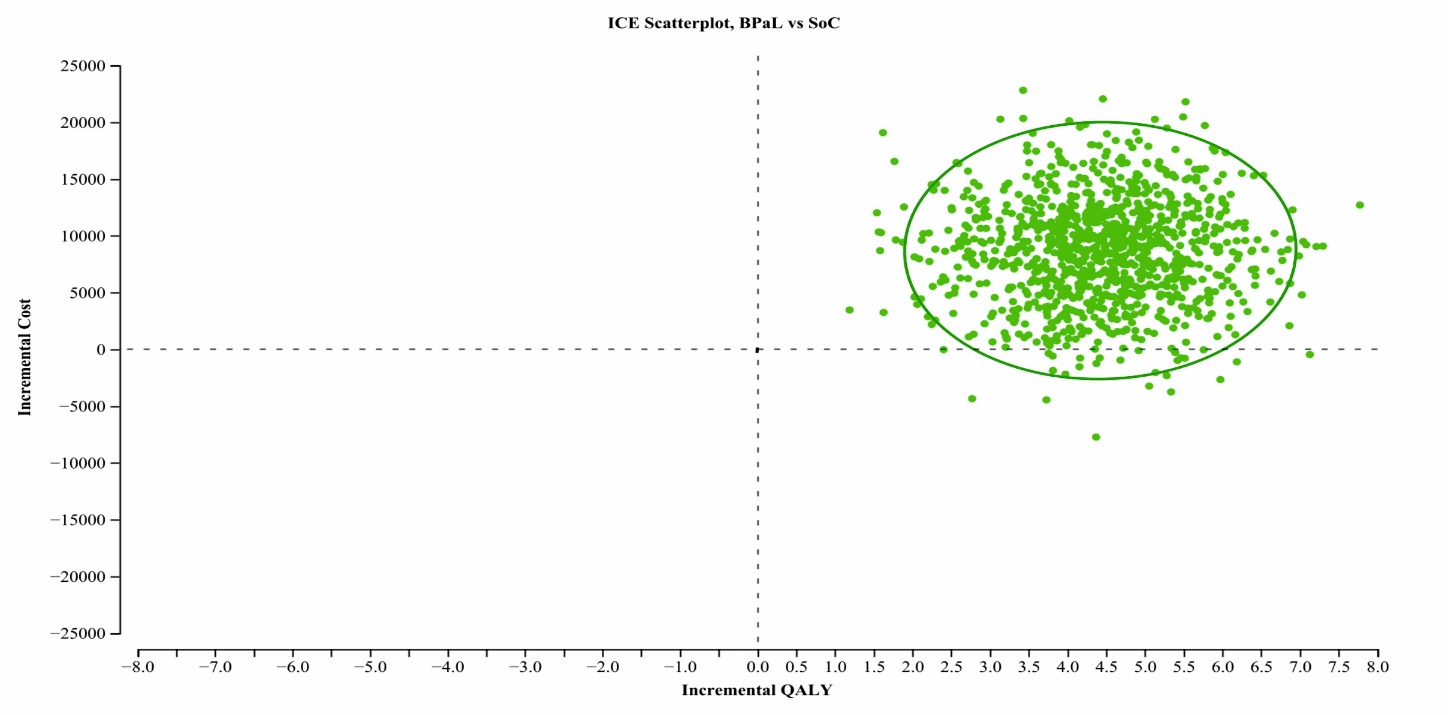
Figure S9.** BPaL with current SoC PSA joint incremental cost and QALY

**Figure S10.** BPaLM with current SoC PSA joint incremental cost and QALY

**
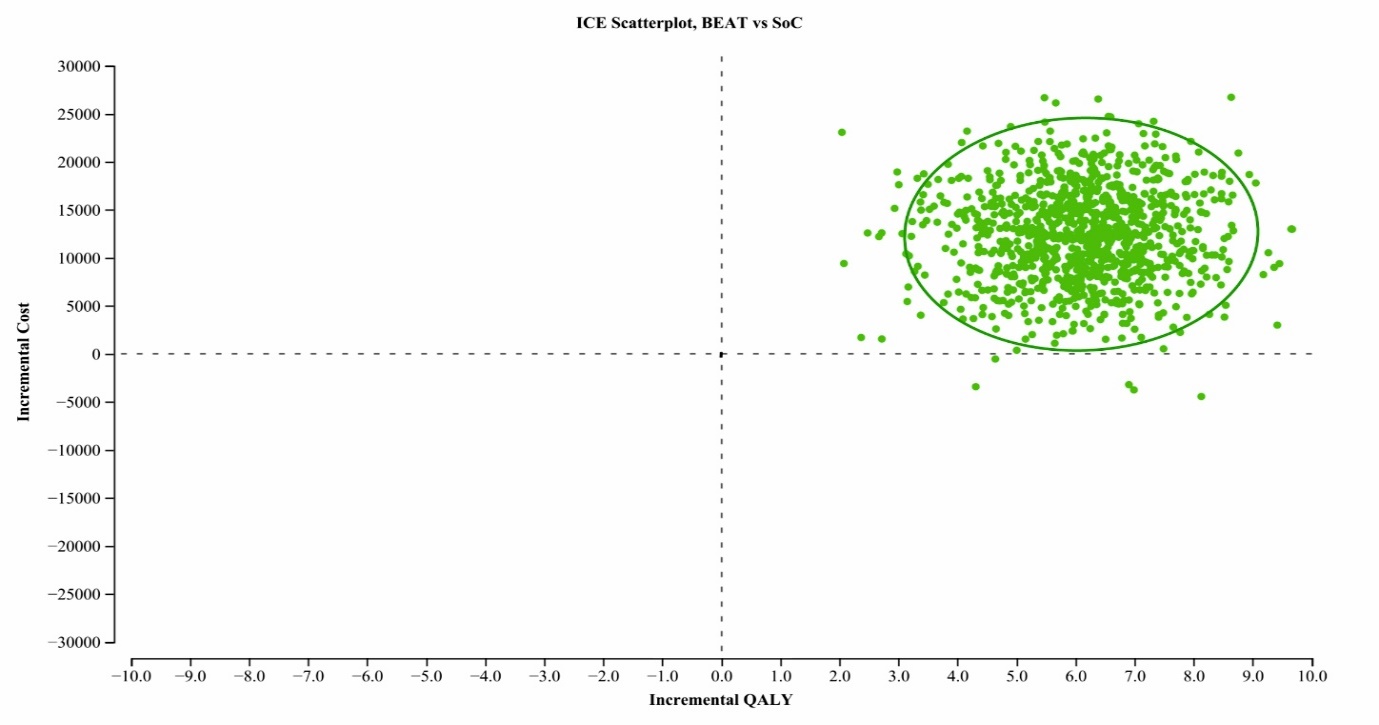
**


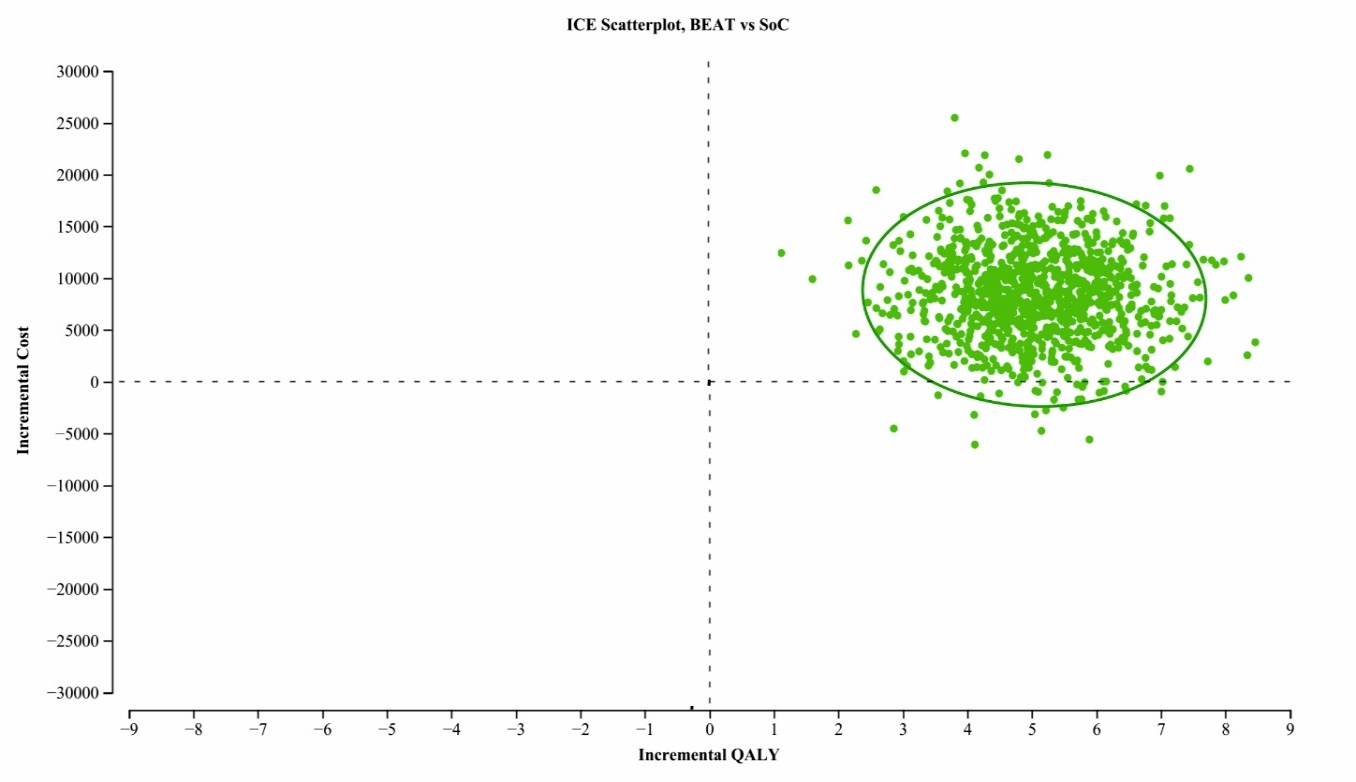
**Figure S11.** BPaLC with current SoC PSA joint incremental cost and QALY

**
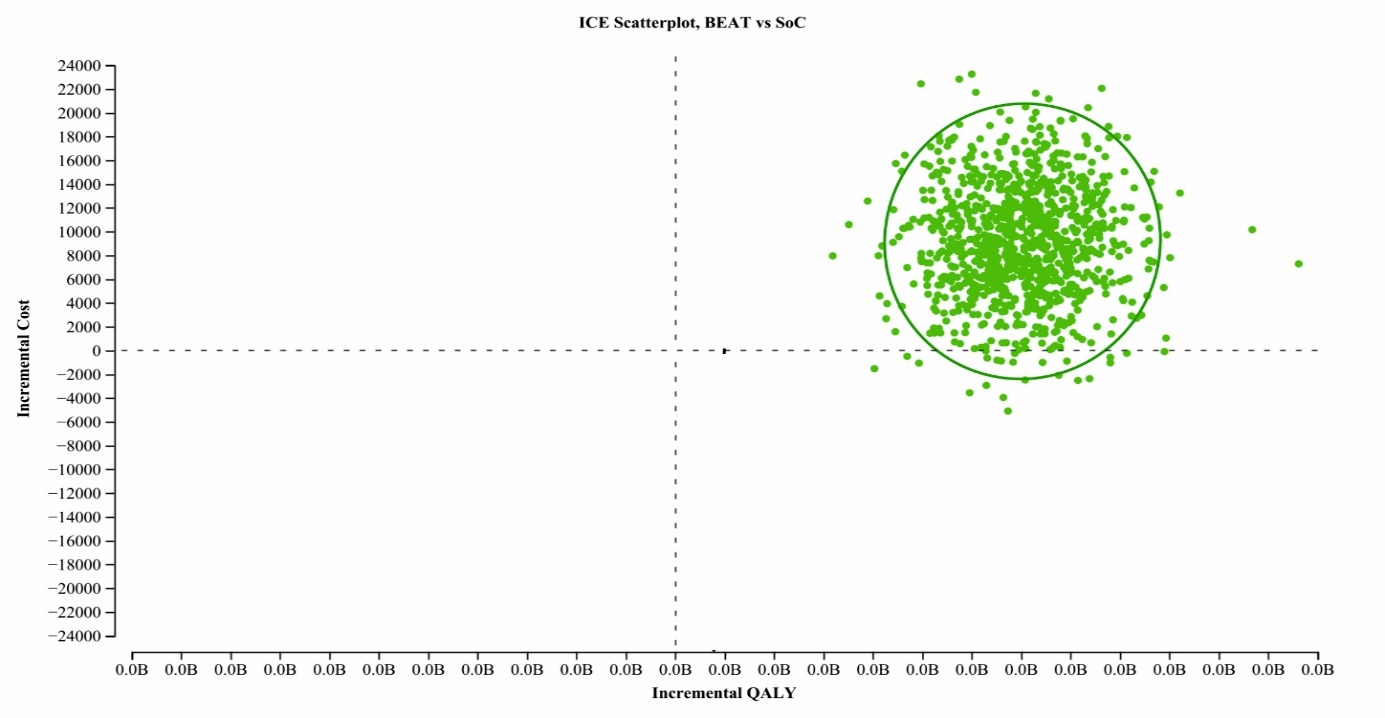
Figure S12.** mBPaL1 with current SoC PSA joint incremental cost and QALY

**Figure S13.** mBPaL2 with current SoC PSA joint incremental cost and QALY


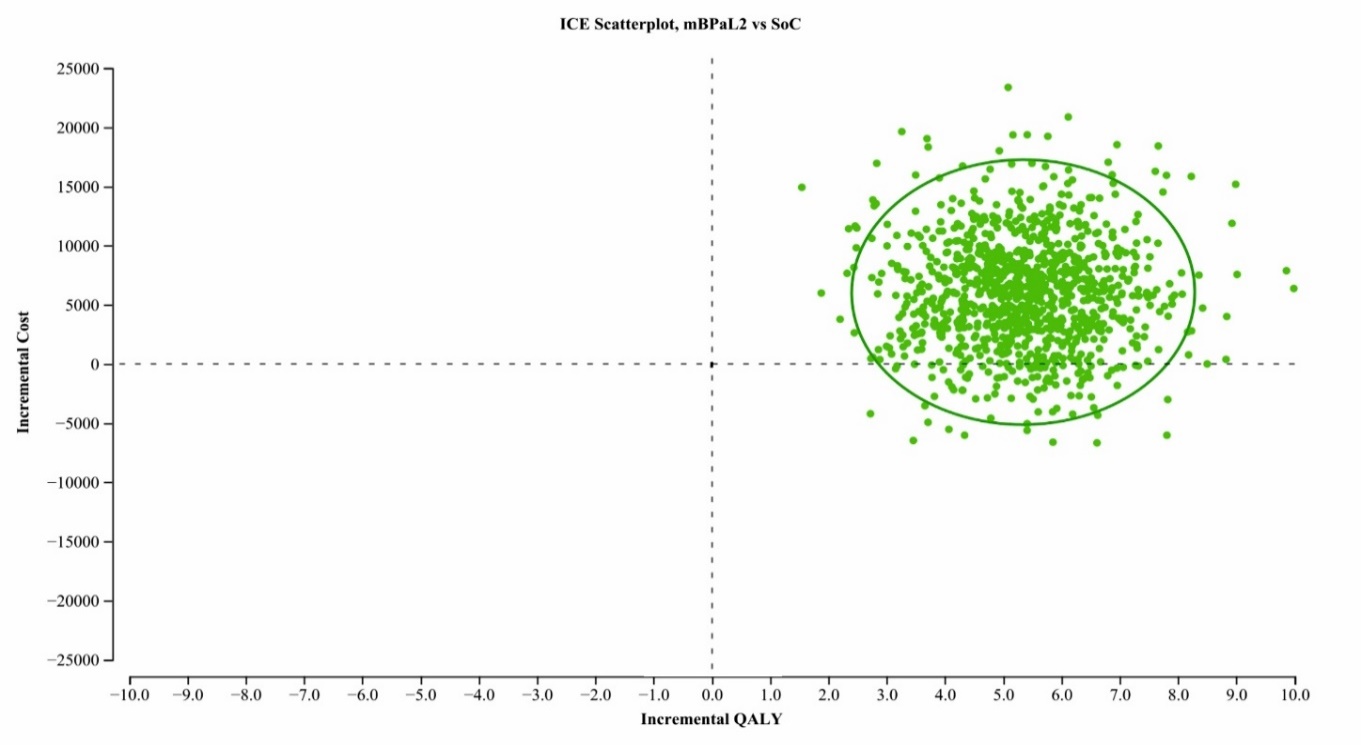


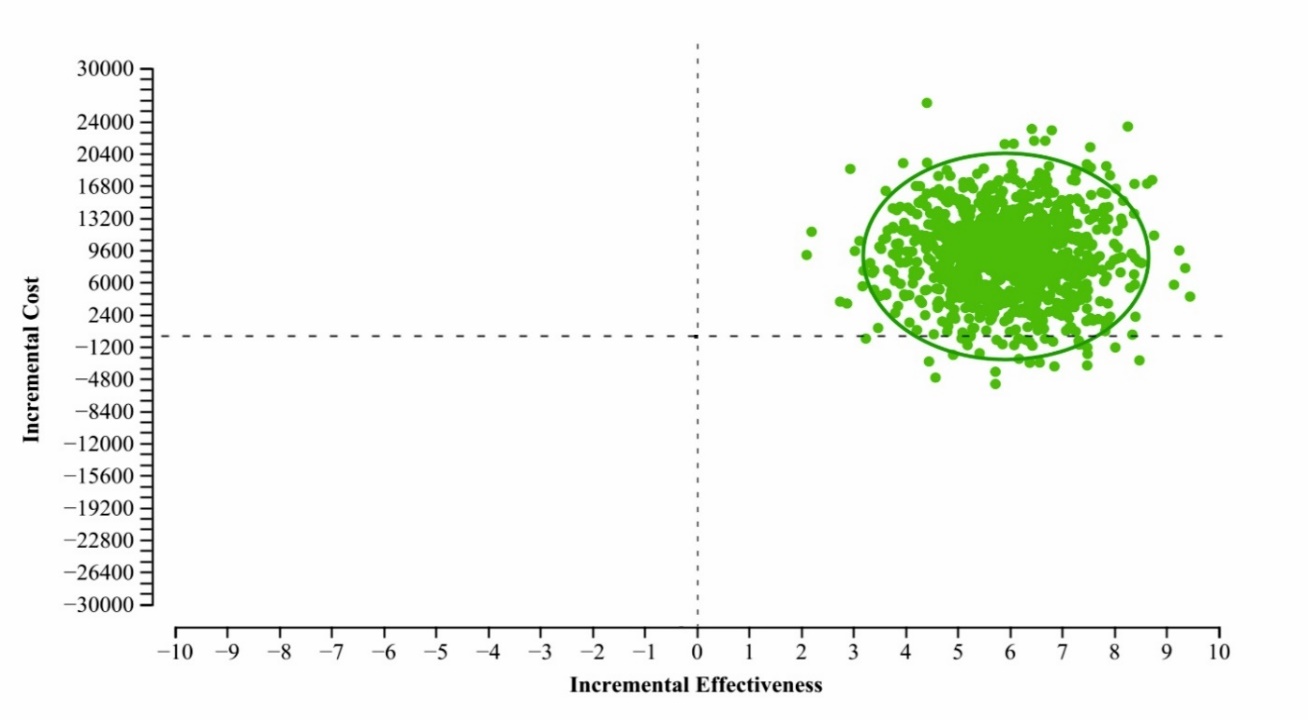
**Figure S14.** mBPaL3 with current SoC PSA joint incremental cost and QALY

**
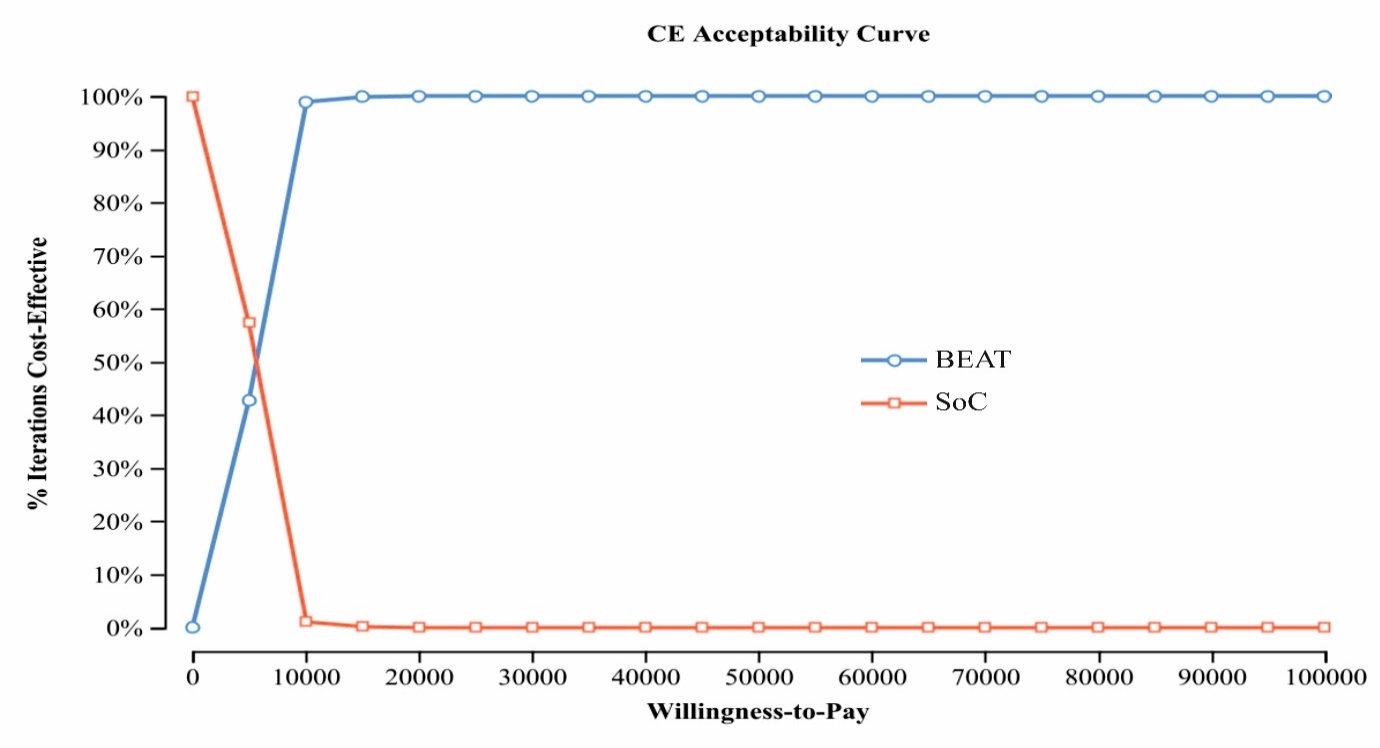
Figure S15.** CEAC curve for BEAT with current SoC

**
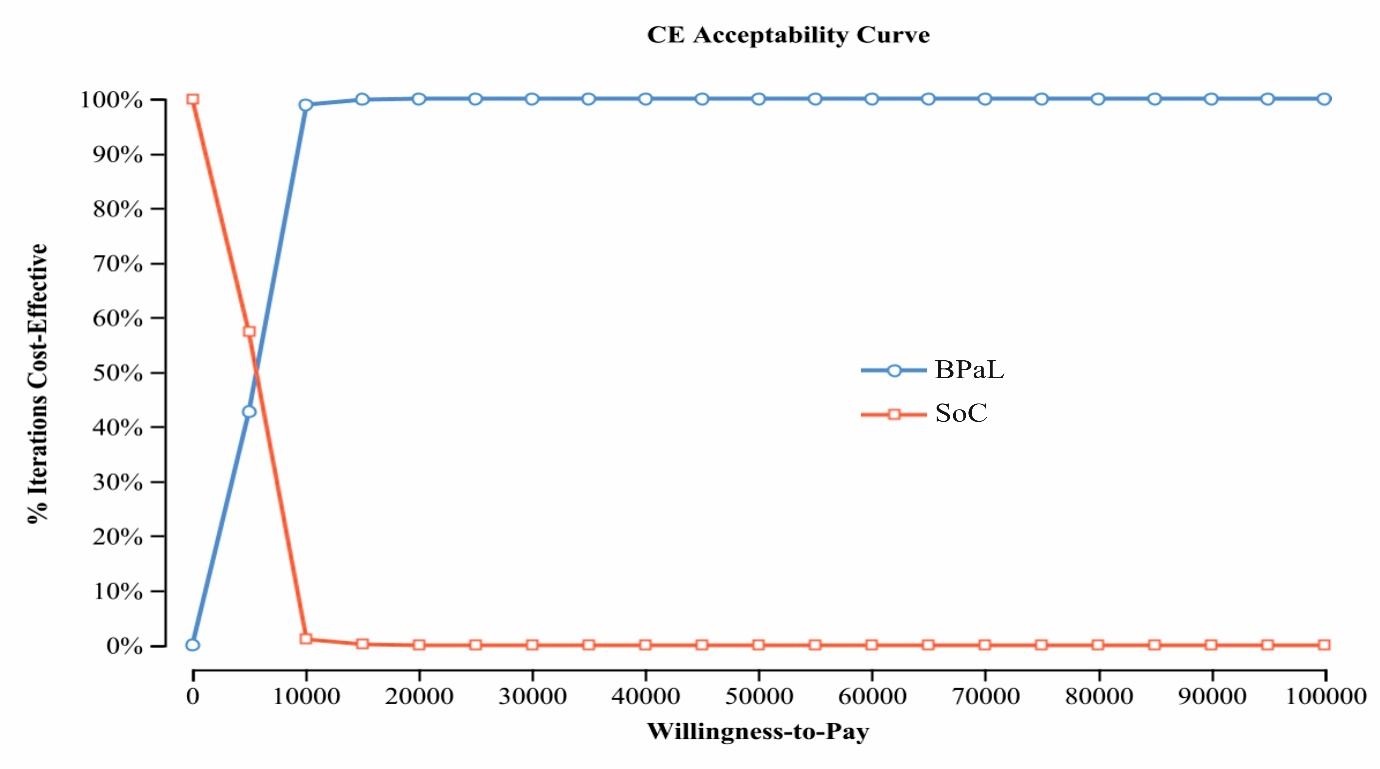
Figure S16.** CEAC curve for BPaL with current SoC

**Figure S17.** CEAC curve for BPaLM with current SoC

**
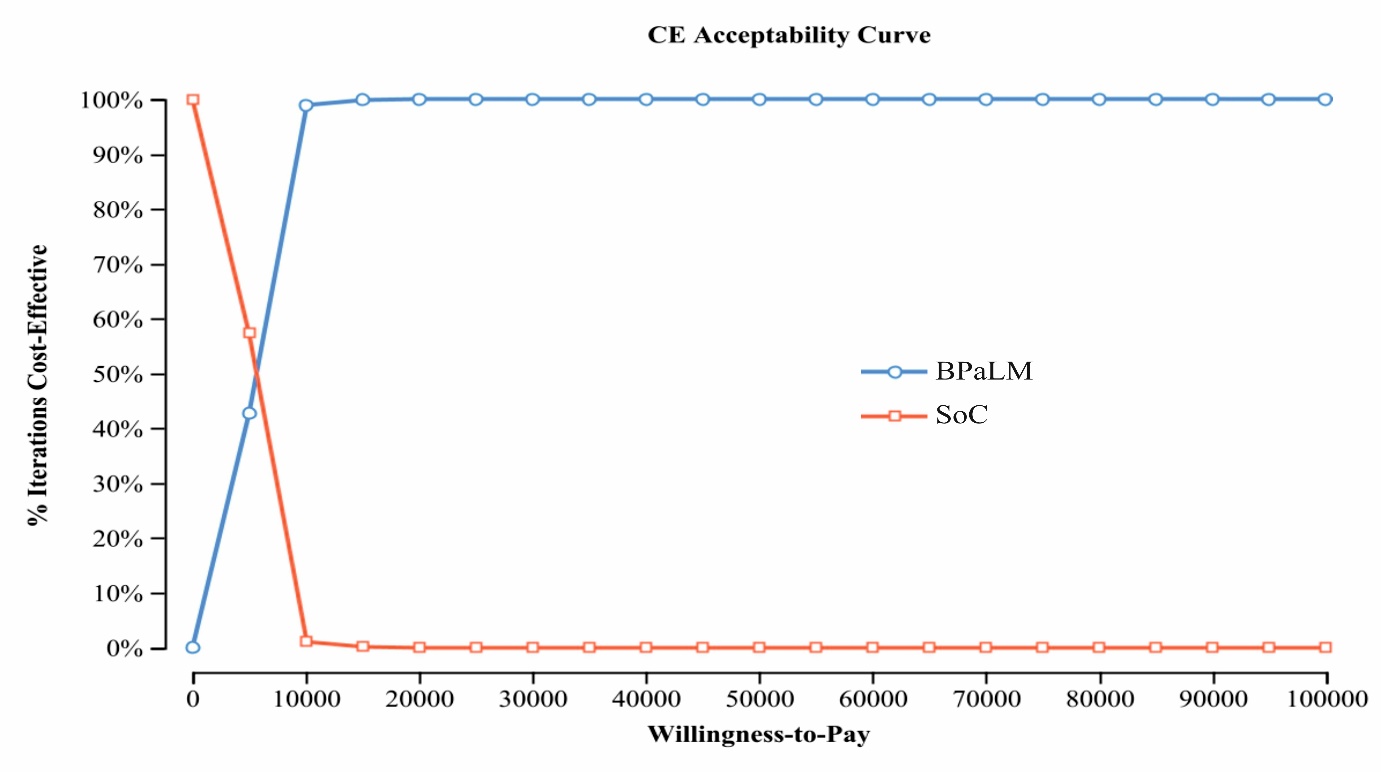
**

**
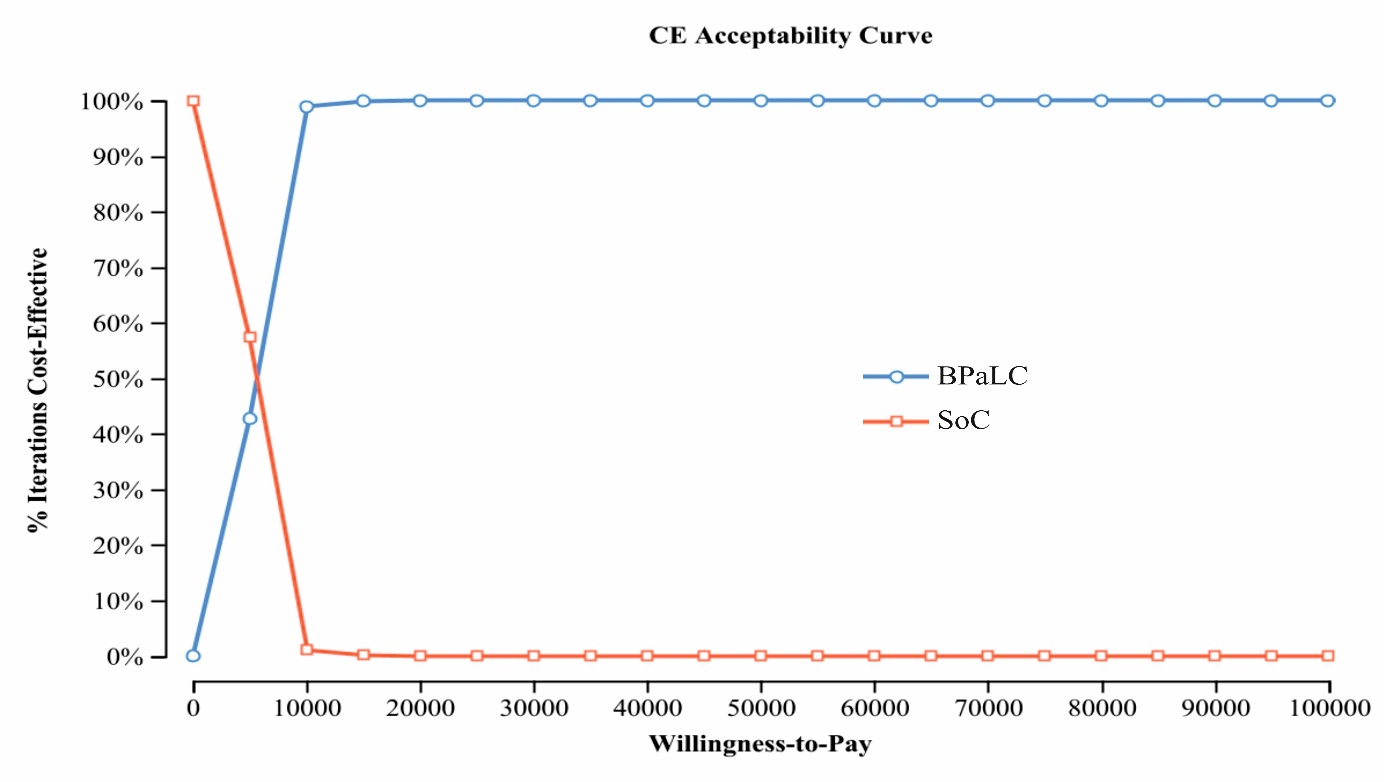
Figure S18.** CEAC curve for BPaLC with current SoC

**
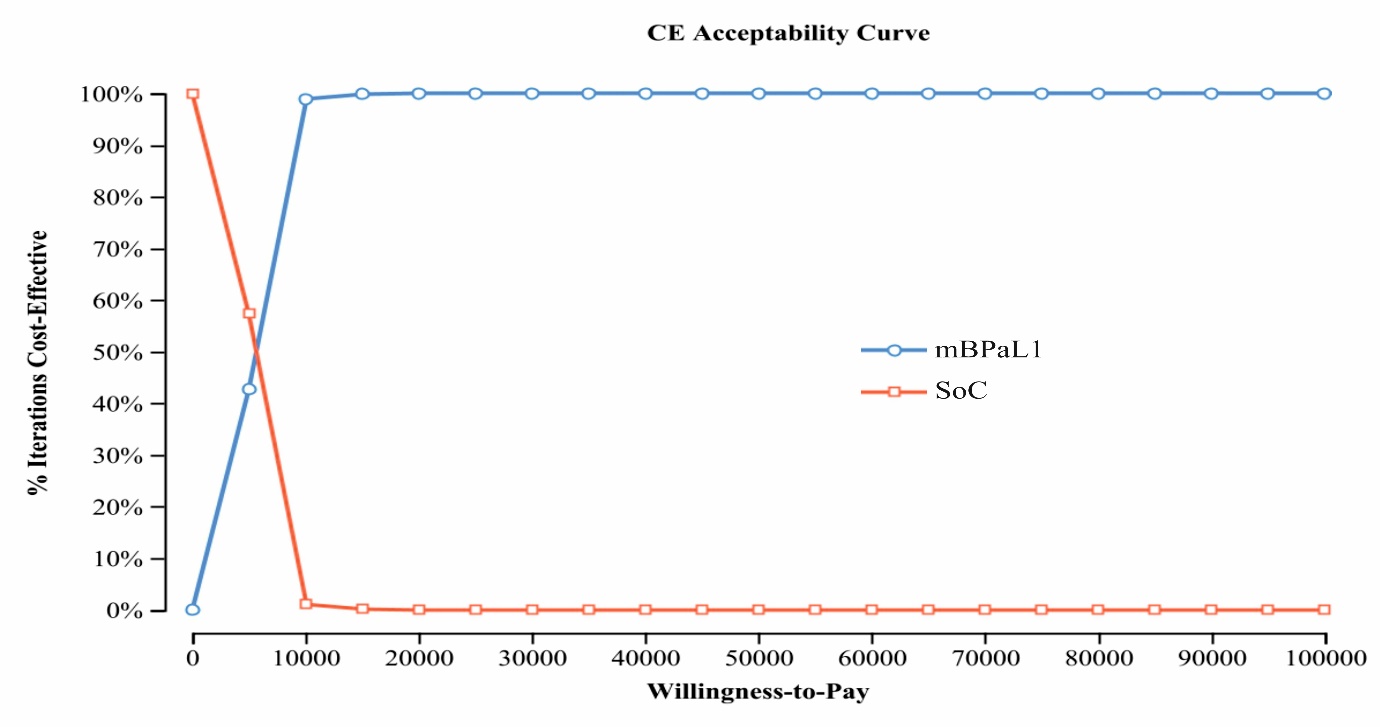
Figure S19.** CEAC curve for mBPaL1 with current SoC

**
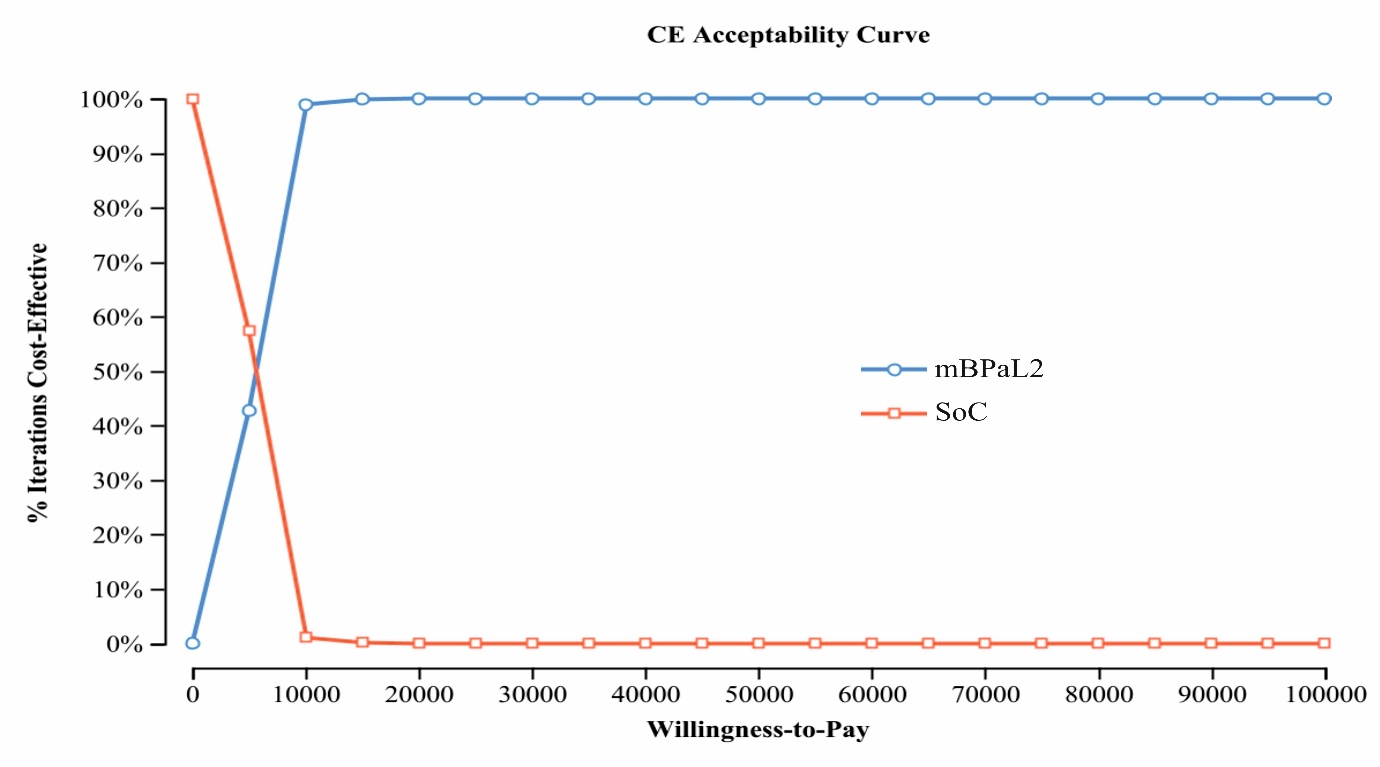
Figure S20.** CEAC curve for mBPaL2 with current SoC

**
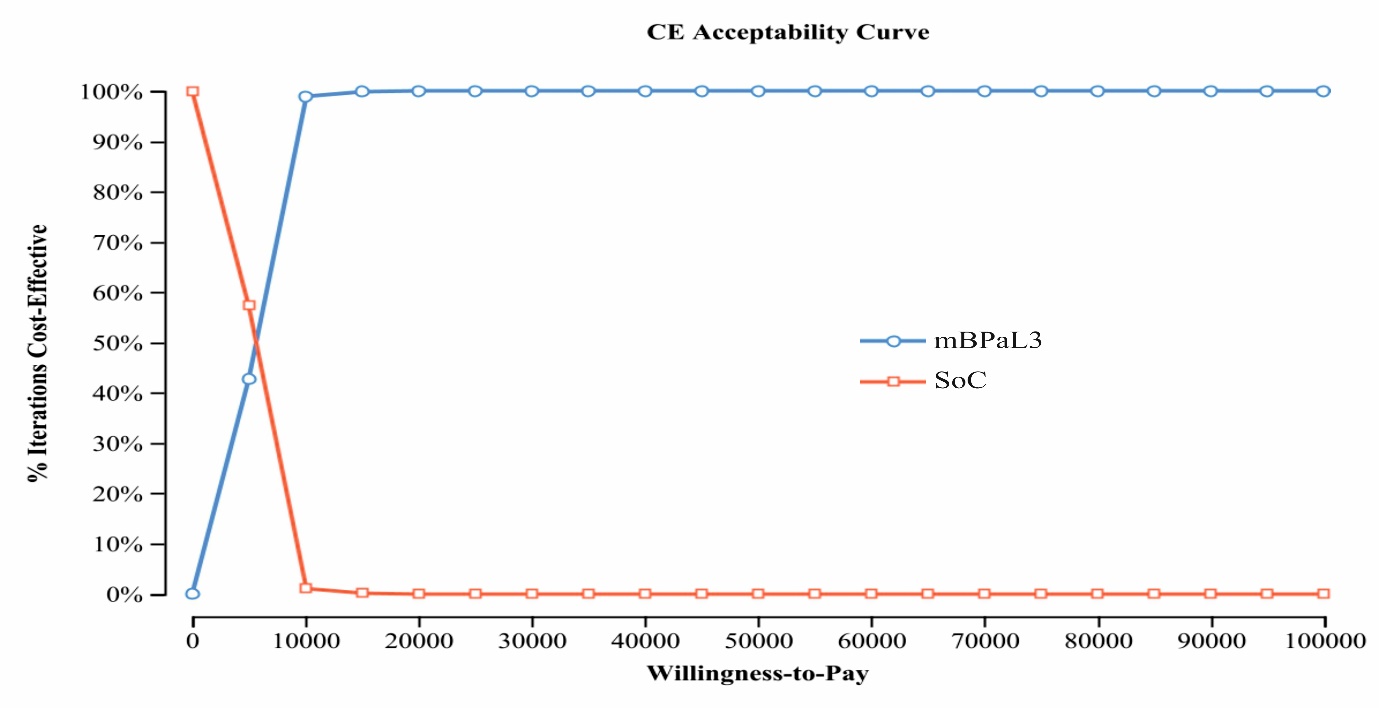
Figure S21.** CEAC curve for mBPaL3 with current SoC

**TECHNICAL NOTES**

**Definitions**

**Tuberculosis (TB) disease**

A disease in humans caused by the M. tuberculosis complex, which comprises eight distinct but closely related organisms – M. bovis, M. caprae, M. africanum, M. microti, M. pinnipedii, M. mungi, M. orygis and M. canetti. The most common and important agent of human disease is M. tuberculosis (1).

**Drug Resistant Tuberculosis (DR-TB)**

Tuberculosis (TB) disease caused by a stain of Mycobacterium tuberculosis complex that is resistant to any TB medicines (1).

**Multidrug-resistant TB (MDR-TB)**

TB disease caused by a strain of M. tuberculosis complex that is resistant to rifampicin and isoniazid (1).

**Rifampicin-resistant TB (RR-TB)**

TB disease caused by a strain of M. tuberculosis complex that is resistant to rifampicin. These strains may be susceptible or resistant to isoniazid (i.e. multidrug resistant TB [MDR-TB]), or resistant to other first-line or second-line TB medicines (1).

**MDR/RR-TB**

MRR/RR –TB refers to either multidrug-resistant TB (MDR-TB) or rifampicin-resistant TB (RR-TB) (1).

**Extensively drug-resistant TB (XDR-TB)**

TB disease caused by a strain of M. tuberculosis complex that is resistant to rifampicin (and may also be resistant to isoniazid), and that is also resistant to at least one fluoroquinolone (levofloxacin or moxifloxacin) and to at least one other “Group A” drug (Bedaquiline or linezolid) (1).

**Pre-extensively drug-resistant TB (pre-XDR-TB)**

TB disease caused by a strain of M. tuberculosis complex that is resistant to rifampicin (and may also be resistant to isoniazid), and that is also resistant to at least one fluoroquinolone (either levofloxacin or moxifloxacin) (1).

**Adverse Drug Reaction (ADR)**

A response to a drug that is noxious and unintended and occurs at doses normally used in man for the prophylaxis, diagnosis or therapy of disease, or for modification of physiological function (2). The ADR in this study includes no ADR, normal ADR and serious ADR cases as it requires hospitalization.

**Serious adverse event (Serious ADR)**

An adverse event that leads to death or a life-threatening experience, to hospitalization or prolongation of hospitalization, to persistent or significant disability, or to a congenital anomaly. Adverse events that do not immediately result in one of these outcomes but that require an intervention to prevent such an outcome from happening are included. Serious adverse events may require a drastic intervention, such as termination of the drug suspected of having caused the event (1).

**Short course regimens**

The complete course of the treatment period (6-9 months) is shorter than current standard of care which is 9-11 months.

**Culture Conversion**

Sputum culture conversion is defined as bacteriological improvement cultures at the end of the fourth month for the short course regimens and current standard of care (9-11 month) regimen.

**Treatment Extension**

At the end of the treatment, if there are no bacteriological changes in the culture the treatment will be extended further for the next three months in short course regimens. Similarly, it will be extended further for the next 5 months in current standard of care (9-11 months) regimen.

**Cure**

The participant with DR-TB patient who has bacteriologically confirmed DR-TB at the beginning of treatment and has documented bacteriological improvement culture at the last month of treatment and on at least one previous occasion (3).

**Treatment failure**

Treatment failure is defined as the patient who has no bacteriological improvement culture in the fourth and last month of treatment or later after the initiation of the treatment for tuberculosis.

**Lost to follow up**

In this study, patients with sputum culture positive DR-TB whose treatment was interrupted for two or more consecutive months are referred to as loss to follow up.

**Death**

Death is when a TB patient dies of any cause after enrolment into the study and before treatment completion.

**Health system cost**

Health system cost refers to the cost incurred by the provider. The health system in this study is a public health facility that provides medical services at a subsidized rate or free of cost. Thus all medical cost was assumed to be incurred only by the public health facility. This includes Human resources, Equipment, Investigation, Treatment, and Medication cost.

**Time Horizon**

When designing comparative outcomes or cost-effectiveness analysis, the time horizon defining the duration of time for outcomes assessment is considered. The time horizon must be long enough to capture the intended and unintended benefits and harms of the intervention. This study considers a particular time point horizon to capture all costs and effectiveness.

**Decision Tree**

A Decision tree is a flowchart like a tree structure, where each internal node denotes a test on an attribute, each branch represents an outcome of the test and each leaf node (terminal node) holds a class label.

**Cost-effectiveness analysis**

Cost-effectiveness analysis (CEA) is a way to examine both the costs and health outcomes of one or more interventions. It compares an intervention to another intervention (or the status quo) by estimating how much it costs to gain a unit of a health outcome like a life-year gained or death prevented.

**Incremental cost-effectiveness ratio**

The cost-effectiveness of short course regimens was assessed by calculating the ICER between the proposed strategies and the current strategy. The difference in costs and QALYs of the proposed strategies and the current strategy was used to calculate ICER using the following formula.

$$ICER=\frac{Total cost ofproposed strategy-Total cost of current strategy}{Total QALY of proposed strategy-Total QALY of current strategy}$$

**Quality Adjusted Life Year**

The Quality-Adjusted Life Year (QALY) is a standardized measure of disease burden which combines both survival and health-related quality of life into a single index. The QALY is primarily used in cost-effectiveness analyses to guide decisions regarding the distribution of limited health care resources among competing health programs or interventions for a population of interest but has also been used to aid decisions regarding clinical management and individual patient care.

$$QALY= Utility * Expected Life years$$

**One-way sensitivity analysis**

Univariate/one-way sensitivity analysis (OWSA) is to assess the impact that changes in a certain input (parameter) will have on the output results of an economic evaluation. This will help to assess the robustness of the result to that parameter. It is helpful for decision-makers to have insights into the relationship between specific input parameters and the model outputs.

**Probabilistic sensitivity analysis**

Probabilistic sensitivity analysis (PSA) demonstrates the parameter uncertainty in a decision problem. The technique involves sampling parameters from their respective distributions (rather than simply using mean/median parameter values). This technique used in economic modeling allows the modeler to quantify the level of confidence in the output of the analysis, concerning uncertainty in the model inputs.

**Cost estimation**

Costs were obtained from the published literature. The staff incentive cost, food cost, and travel cost for a standard eighteen-month regimen and six-month regimen were obtained from RBIPMT and NTEP. Average hospitalization cost per patient due to adverse drug reaction was obtained from literature and was considered similar in both the arm assuming that it is similar in both treatment regimens.

**Formulae:**

1. Lower value

$$(Mean value)*(1-OWSA\_Range)$$

2. Upper value

$$(Mean value)*(1+OWSA\_Range)$$

3. Standard error

$$\frac{\left( Upper value-lowe Value \right)}{(2*NORMSINV \left( 0.975 \right))}$$

4. Beta Distribution

Alpha

$$(Mean value)*(Mean value*\frac{\left( 1-Mean value \right)}{{(Standard error}^{2})}-1)$$

Beta

$$(1-Mean value)*(Mean value*\frac{\left( 1-Mean value \right)}{{(Standard error}^{2})}-1)$$

1. Gamma distribution

Alpha

$${(\frac{Mean value}{Standard error})}^{2}$$

Beta

$${\frac{(Standard error)}{Mean value}}^{2}$$

1. log Normal distribution

Alpha

$$LN \left( \$Mean value \right)-\frac{{(\$beta of\log normal distribution)}^{2}}{2}$$

Beta

$$\sqrt{\frac{LN(1+\$Standard erro{r)}^{2}}{{(\$Mean value)}^{2}}}$$

1. Normal distribution

Alpha

$$\left( \$Mean value \right)-\frac{{(\$beta of normal distribution)}^{2}}{2}$$

Beta

$$\sqrt{\frac{(1+\$Standard erro{r)}^{2}}{{(\$Mean value)}^{2}}}$$

1. For a Cost-effective analysis curve:

SERIES (PSA! (Select the 1000 iterations of Cost-Effective treatment, PSA! select the 1000 iterations of Cost-Effective%, 1)

1. For cost-effectiveness plane:

SERIES (PSA! select the regimen heading, select the 1000 iteration of PSA Joint incremental QALYs, PSA! select the 1000 iteration of PSA Joint Incremental Cost, 1)

1. For all the cost parameters we multiplied per patient cost into the cohort population

For example:

Cost of food per patient is 300 and Cohort population is 100000, we calculate it as

$300*100000=$ 30000000

**References**

[1] WHO consolidated guidelines on tuberculosis. Module 4. 2022 updated <https://www.who.int/publications/i/item/9789240063129>

[2] International drug monitoring: the role of national centres Report of a WHO meeting. World Health Organ Tech Rep Ser. 1972; 498:1–25.

[3] Guidance for National Tuberculosis Programmes on the Management of Tuberculosis in Children. (2014). (2nd ed.). World Health Organization.
