# Supplementary material for: Comparing cost-effectiveness of short-course regimens for drug-resistant tuberculosis treatment in India
Source: Int J Technol Assess Health Care. 2025 Jul 21;41(1):e48. doi: 10.1017/S0266462325100329 (PMC12322854; doi:10.1017/S0266462325100329)
Supplement: Muniyandi et al. supplementary material 2 — Muniyandi et al. supplementary material [file S0266462325100329sup002.docx]

**Manuscript Number**: IJTAHC-2025-0002

**Title of the manuscript:** Comparing Cost-Effectiveness of Short Course Regimens for Drug-Resistant Tuberculosis Treatment in India

**DR-TB Model TreeAge Files Link:**

Access Link <https://drive.google.com/file/d/1EgozzIan5sImhGmqx8Krb_mOwpA1rxHd/view?usp=drive_link>
